# Comparative analysis of the NLR gene family in the genomes of garden asparagus (*Asparagus officinalis*) and its wild relatives

**DOI:** 10.3389/fpls.2025.1681919

**Published:** 2025-09-25

**Authors:** Li-Ping Sun, Wen-Zhuo Zhai, Rui-Yan Song, Hao-Han Ning, Shu-Fen Li, Wu-Jun Gao

**Affiliations:** ^1^ College of Life Sciences, Henan Normal University, Xinxiang, China; ^2^ School of Biological Engineering, Xinxiang University, Xinxiang, China; ^3^ Henan Engineering Technology Research Center for Conservation and Utilization of Genuine Medicinal Herbs, Xinxiang, China

**Keywords:** garden asparagus, NLR gene family, evolution, disease resistance, genome-wide comparative analysis

## Abstract

Garden asparagus (*Asparagus officinalis*), a prominent horticultural crop recognized as the “king of vegetables” in the international market, is usually threatened by severe disease challenges that hinder its sustainable cultivation. Nucleotide-binding leucine-rich repeat receptors (NLRs) are key components of plant immune systems, yet their specific role and evolutionary patterns in *A. officinalis* and its related species remain poorly characterized. In this study, we conducted a comprehensive analysis of NLR gene distribution patterns, structural features, phylogenetic characterization, and evolutionary dynamics across *A. officinalis* and two phylogenetically related species, *Asparagus kiusianus* and *Asparagus setaceus*, and conducted expression studies after *Phomopsis asparagi* infection in *A. officinalis*. Our findings demonstrate that NLR genes in all three species display chromosomal clustering patterns. Phylogenetic reconstruction and N-terminal domain classification categorized these NLRs into three distinct subfamilies, with their promoters containing numerous cis-elements responsive to defense signals and phytohormones. Comparative genomic analysis revealed a marked contraction of the NLR genes from the wild species to the domesticated *A. officinalis*, with gene counts of 63, 47, and 27 NLR genes identified in *A. setaceus*, *A. kiusianus*, and *A. officinalis*, respectively. Orthologous gene analysis identified 16 conserved NLR gene pairs between *A. setaceus* and *A. officinalis*, which are likely the NLR genes preserved during the domestication process of *A. officinalis*. Pathogen inoculation assays revealed distinct phenotypic responses: *A. officinalis* was susceptible, while *A. setaceus* remained asymptomatic. Notably, the majority of preserved NLR genes in *A. officinalis* demonstrated either unchanged or downregulated expression following fungal challenge, indicating a potential functional impairment in disease resistance mechanisms. In conclusion, our findings suggest that the increased disease susceptibility of domesticated *A. officinalis* is driven by both the contraction of NLR gene repertoire and the functional reduced or inconsistent induction of retained NLR genes—potentially a consequence of artificial selection favoring yield and quality. This study provides important insights into the evolutionary dynamics of NLR genes within the *Asparagus* genus and may contribute to future efforts aimed at disease-resistant breeding in *A. officinalis*.

## Introduction

Garden asparagus (*Asparagus officinalis*), a prominent horticultural crop, is esteemed as the “king of vegetables” and ranks among the world’s top ten vegetable crops in the international market due to its high nutritional and health benefits (Harkess et al., 2017). However, in commercial cultivation, its growth and development are severely affected by fungal diseases such as brown spot, leaf blight, and stem blight (Sun et al., 2024). The close wild relatives often harbor abundant resistance resources. Previous studies have demonstrated that the disease resistance of wild asparagus species *Asparagus kiusianus* is superior to that of garden asparagus. Furthermore, *A. kiusianus* can hybridize with *A. officinalis* to produce fertile offspring, with the hybrid progeny demonstrating significantly enhanced resistance to asparagus stem blight (Ito et al., 2011; Abdelrahman et al., 2017). These wild species represent valuable genetic resources for resistance breeding, yet comparative analyses of resistance (R) genes across the genomes of these species remain insufficient. Currently, within the *Asparagus* genus, the genomes of *A. officinalis*, *Asparagus setaceus*, and *A. kiusianus* have been sequenced, providing a foundational dataset for systematic comparisons of their resistance genes.

Plants possess an extensive repertoire of R genes that confer protection against diverse pathogens. The most prominent class of R genes encodes nucleotide-binding leucine-rich repeat receptors (NLRs), which are characterized by three conserved domains: an N-terminal domain, a central nucleotide-binding site (NBS) domain, and a C-terminal leucine-rich repeat (LRR) region (Eitas and Dangl, 2010; Lee and Yeom, 2015). Based on their N-terminal domains, NLRs are classified into distinct subfamilies: CNLs (containing CC domains), TNLs (with TIR domains), and RNLs (featuring RPW8 domains) (Shao et al., 2016; Jubic et al., 2019). Although full-length NLRs contain all three domains, truncated variants lacking specific domains (e.g., NL, CN, RN, TN, or N) retain their functional classification (Steele et al., 2019). The central NB-ARC domain contains conserved motifs, including the P-loop, GLPL, MHD, and Kinase 2, which are critical for immune function (Steele et al., 2019).

Comparative genomic analyses across various plant species have identified NLRs as the most variable gene family, likely due to pathogen-driven selection pressures (Seo et al., 2016), For example, within the Poaceae family, *Oropetium thomaeum* harbors several dozen NLRs, whereas *Triticum aestivum* contains over two thousand (Liu et al., 2021). Typically, CNLs and TNLs exhibit more rapid expansion compared to RNLs, with the former often reaching hundreds per genome, in contrast to the single-digit counts observed for RNL (Shao et al., 2016; Neupane et al., 2018; Xue et al., 2019). The precise regulation of NLR expression is crucial, as dysregulation can lead to autoimmunity, thereby adversely affecting plant growth and yield (van Wersch et al., 2020).

In this study, we performed a comprehensive genome-wide identification and comparative analysis of NLR genes in the genomes of *A. officinalis*, *A. kiusianus*, and *A. setaceus*. By integrating phylogenetic, evolutionary, transcriptomic, and functional analyses of the NLR family, we elucidate the molecular determinants that contribute to disease susceptibility in cultivated *A. officinalis* and provide a foundation for future resistance breeding programs.

## Materials and methods

### Genome-wide identification, classification, and localization analysis of NLR genes

The genomic and annotation data of *A. officinalis* were generated by our laboratory (unpublished results, BUSCO assessment using the embryophyta_odb10 database shows that the completeness of genome assembly and gene annotation is 97.5% and 98.1%, respectively.). For comparative analyses, we obtained genomic resources for *A. kiusianus* from Plant GARDEN (https://plantgarden.jp) (No:DRA012987) (Shirasawa et al., 2022) and for *A. setaceus* from Dryad Digital Repository (https://doi.org/10.5061/dryad.1c59zw3rm) (Li et al., 2020).

We employed a dual approach for comprehensive NLR identification. First, Hidden Markov Model (HMM) searches were performed using the conserved NB-ARC domain (Pfam: PF00931) as query. Second, local BLASTp analyses (BLAST+ v2.0) were conducted against reference NLR protein sequences from *Arabidopsis thaliana*, *Oryza sativa*, and *Allium sativum*, applying a stringent E-value cutoff of 1e-10. Candidate sequences identified through both methods were extracted using TBtools (Chen et al., 2020) and subsequently validated through domain architecture analysis.

Protein domains were characterized using InterProScan (Jones et al., 2014) and NCBI’s Batch CD-Search, with sequences containing the NB-ARC domain (E-value ≤ 1e^-5^) retained as bona fide NLR genes. Final classification was performed by querying the Pfam (http://pfam.xfam.org/) and PRGdb 4.0 (http://prgdb.org/prgdb4/plants/?id=590262) databases (Mistry et al., 2021), with genes categorized based on their complete domain architecture and chromosomal distribution.

The chromosomal distribution of NLR family members was determined using TBtools v2.136 (Chen et al., 2020), with gene positional information extracted from genome annotations and subsequently visualized through chromosomal mapping (van Wersch et al., 2020).

Sub-cellular localization of the identified NLRs was determined using WoLF PSORT (https://wolfpsort.hgc.jp/) (Horton et al., 2007; Hu et al., 2015). The results were visualized using Python scripts to generate a subcellular localization heatmap.

### Motif and conserved domain analysis of NLR genes

To characterize the domain architecture of the NBS gene family, conserved motifs within NBS domains were predicted using MEME suite (https://meme-suite.org/meme/tools/meme) (Bailey et al., 2015). The analysis was performed with the motif number set to 10 while maintaining default parameters as previously established (Nepal et al., 2017). Resulting motif distributions were visualized using TBtools, and gene structures were subsequently analyzed through GSDS 2.0 (Gene Structure Display Server).

### Prediction of cis-acting regulatory elements

The PlantCARE (http://bioinformatics.psb.ugent.be/webtools/plantcare/html/) (Lescot et al., 2002) was utilized to analyze the cis-acting regulatory elements of the NBS-LRR gene’s promoter sequence (2000 bp genome sequence upstream of the initial codon ATG). Their distribution and heat map were plotted using TBtools.

### Phylogenetic analysis of NBS encoding genes in *A. officinalis* and its closely related species

The protein sequences of candidate NLR genes from *A. officinalis* and its closely related species were consolidated into a single file, and multiple sequence alignment was performed using Clustal Omega (Sievers and Higgins, 2018) to generate the aligned file. The phylogenetic tree was constructed by using the maximum likelihood method based on the JTT matrix-based model implemented in MEGA (Yang, 2007). The tree with the highest log likelihood was selected, the nodes were selected, and the nodes were tested 1000 times by Bootstrap analysis.

### Cluster analysis of *A. officinalis* NLRs

Adjacent NLR pairs separated by ≤ 8 genes were retrieved from the genomes of all three species, and their relative orientations (head-to-head, head-to-tail, or tail-to-tail) were determined with BEDTools. Statistical significance was evaluated by χ² tests against random expectations (10–000 permutations).

### Orthogroup analysis of the NLR gene family between *A. officinalis* and *A. setaceus*

For comparison, we used OrthoFinder v2.2.7 (Emms and Kelly, 2019) to cluster orthologous genes of NLR from *A. officinalis* and *A. setaceus* by sequence similarity, the BLAST bit scores were normalized based on gene length and phylogenetic distance.

### Collinearity analysis

“One Step MCScanX” from Tbtools (Chen et al., 2020) was used to perform comparisons across species (*A. officinalis*, *A. kiusianus*, and *A. setaceus*) as well as among common NLR genes and its upstream/downstream genes between *A. officinalis*, and *A. setaceus* to obtain gene pairs and analyze collinear blocks. Non-synonymous (ka) and synonymous (ks) substitution of the paired NLR genes were calculated using Ka Ks_Calculator 2.0 (Wang et al., 2010). Gene duplication events were approximately dated according to the eq. T =Ks/2λ (λ=6.5×10^-9^) (Yu et al., 2005). For *A. setaceus* and *A. officinalis*, NLRs duplication events were detected by MCScanX (Wang et al., 2012), and visualized via Advanced Circos software in TBtools.

### RNA-seq analysis

The comparative transcriptome data generated in a previous study (Abdelrahman et al., 2017) were download from NCBI BioProject (https://www.ncbi.nlm.nih.gov/bioproject/PRJNA591696). Clean reads were mapped to reference *A. officinalis* genome using Salmon (Patro et al., 2017). The FPKM (fragments per kilobase of exon per million reads mapped) and differentially expressed genes were calculated using DESeq2 (Love et al., 2014). Genes with *P* < 0.05 and log_2_ |fold-changes| >1 were considered as DEGs.

### Plant materials, pathogen inoculation and NLR gene expression analysis

*A. officinalis* plants were cultivated and subjected to fungal inoculation following established protocols (Sun et al., 2024). Control plants received sterile distilled water treatment under identical environmental conditions. Stem tissues were harvested at 1-, 2-, and 3-days post-inoculation (dpi) for molecular analyses. Validation of NLR genes expression was performed using reverse transcription quantitative polymerase chain reaction (RT–qPCR). Total RNA was isolated from collected tissues using the RNA Isolation Kit (Vazyme, RC113-01), followed by cDNA synthesis. Quantitative reverse transcription PCR was performed using 2× M5 HiPer SYBR Premix EsTaq (Thermo Fisher Scientific, MF787-02) on a LightCycler480 System (Roche). The *EF1α* gene served as the endogenous reference for normalization, with relative expression levels calculated via the 2^−ΔΔCt^ method. Data in the graph are presented as mean ± standard deviation (n = 3 biological replicates). The ΔCt value for each biological replicate was derived from the mean of its three technical replicates, and the standard deviation was incorporated into the error calculation of the final relative expression values following error propagation rules. Statistical significance (*P* < 0.01) was determined by one-way analysis of variance (ANOVA) followed by Tukey’s *post-hoc* test, with different letters denoting significant differences. Primer sequences are provided in **Supplementary Table S1**.

## Results

### Identification and characterization of the NLR gene family in the genomes of *A. officinalis*, *A. kiusianus*, and *A. setaceus*

**Figure 1:**
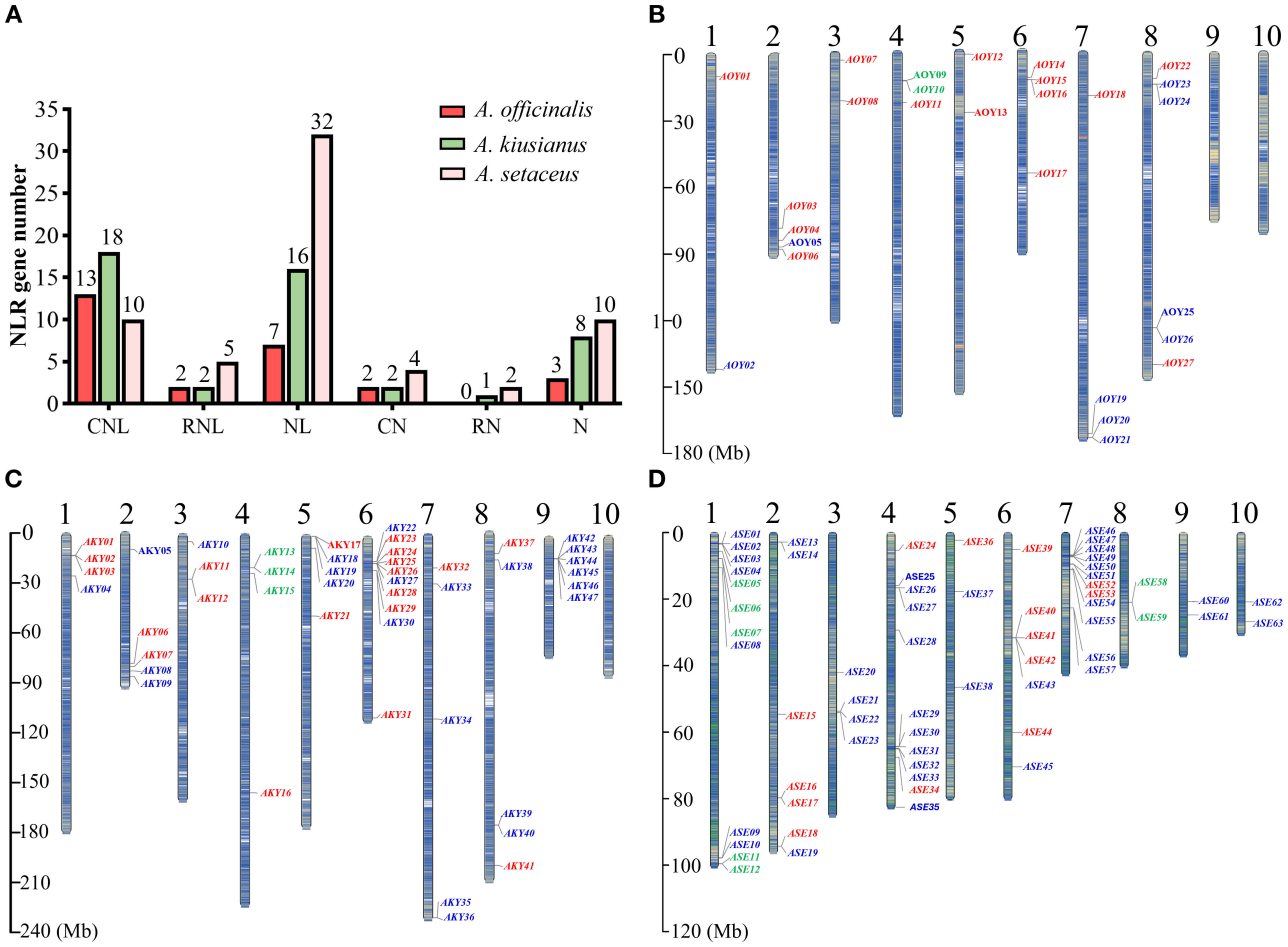
NLR genes number, classification and localization in the chromosomes of *A*. *officinalis*, *A*. *kiusianus*, and *A*. *setaceus*. **(A)** NLR genes number and classification. **(B)** NLR genes localization in the chromosomes of *A*. *officinalis*. **(C)** NLR genes localization in the chromosomes of *A*. *kiusianus*. **(D)** NLR genes localization in the chromosomes of *A*. *setaceus*. CNL-type, RNL-type, and NL-type genes are labeled red, green, and blue, respectively.

A comprehensive genome-wide analysis and manual curation of NLR genes were conducted in the genomes of *A. officinalis*, *A. kiusianus* and *A. setaceus*. Candidate loci were initially retrieved using HMMSEARCH against the NB-ARC Pfam model PF00931, followed by reciprocal BLASTp and domain verification to eliminate false positives. This process resulted in the confident annotation of 27, 47, and 63 NLR genes in *A. officinalis*, *A. kiusianus* and *A. setaceus*, respectively. Based on the presence or absence of canonical N-terminal (CC/RPW8) and C-terminal (LRR) domains, these NBS-encoding genes were categorized into three subfamilies and six classes: (i) CNL subfamily—CC-NBS-LRR (CNL) and CC-NBS (CN); (ii) RNL subfamily—RPW8-NBS-LRR (RNL) and RPW8-NBS (RN); and (iii) NL subfamily—NBS-LRR (NL) and NBS-only (N) (**Figure 1A**). Consistent with the absence of TNL genes in monocots (Guo et al., 2025), no TIR-NBS-LRR members were detected in our analysis. Among the annotated genes, only those containing both the NBS and LRR domains were considered complete NLRs, yielding 22, 36 and 47 loci in *A. officinalis*, *A. kiusianus*, and *A. setaceus*, respectively. The most pronounced variation was observed within the NL subfamily, whose membership increased progressively across the three species (10, 24 and 42 genes, respectively). This pattern suggests recurrent loss of N-terminal domains (CC or RPW8) following duplication events, with *A. setaceus* exhibiting the most extensive truncation since its divergence from the other taxa. Collectively, these data highlight the dynamic evolution of NLR genes and reveal species-specific distribution patterns within three species.

### Chromosomal distribution of NLRs in *A. officinalis*, *A. kiusianus* and *A. setaceus*

To understand the chromosomal distribution of the NLR genes, we generated detailed chromosomal maps of the NLR genes and named according to their locations from top to bottom on the chromosomes (**Supplementary Table S2**). The NLR genes are unevenly distributed across the 10 chromosomes (**Figures 1B–D**). Chromosomes 9 and 10 of *A. officinalis* lack NLR genes, whereas all chromosomes of *A. kiusianus* and *A. setaceus* carry NLR genes. RNL genes are distributed on specific chromosomes. For instance, RNL genes are located on chromosome 4 in *A. officinalis*, and *A. kiusianus*, and chromosomes 1 and 8 in *A. setaceus*. The NLR genes showed preferential clustering at chromosomal termini, with pronounced aggregation in specific genomic regions, potentially reflecting evolutionary selection pressures in these species.

The result of subcellular localization showed that nuclear-localized NLRs predominated (56% and 49% respectively), exceeding cytoplasmic localization (41% and 39%) in *A. officinalis* and *A. kiusianus*. Conversely, *A. setaceus* showed an inverse pattern with 43% cytoplasmic and 31% nuclear localization (**Supplementary Figure S1**). These findings align with emerging evidence that dynamic nucleo-cytoplasmic partitioning of NLR proteins facilitates activation of distinct defense signaling pathways in different cellular compartments (Tameling and Baulcombe, 2007; Wang et al., 2023).

### Conserved domains analysis of NLRs

**Figure 2:**
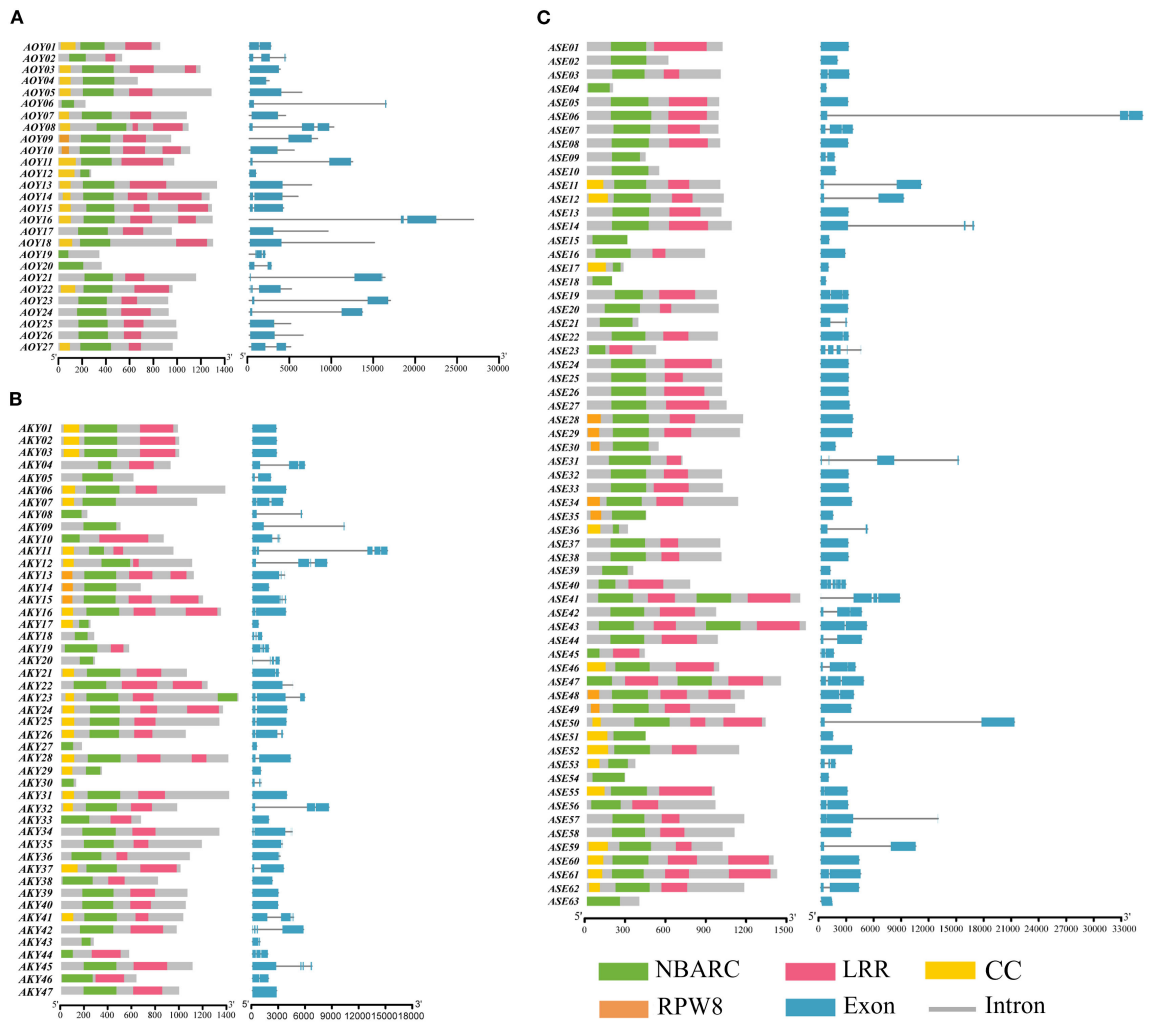
The domain pattern and exon-intron structure of NLR genes in **(A)** *A*. *officinalis*, **(B)** *A*. *kiusianus*, **(C)** *A*. *setaceus*.

Domain analysis using InterProScan and NCBI Batch CD-Search identified four characteristic domains in the NLR repertoire: RX-CC-like (CC), NB-ARC, LRR, and RPW8 (**Figure 2**). While all candidate genes from *A. officinalis* and its related species contained the NB-ARC domain. Among them, with 50 (36.8%) typical proteins showing intact domains, and the rest (86) lacking at least one important domain. 106 genes (77.9% of the total) possessed the LRR domain, 47 genes (34.6%) contained the CC domain, and 13 genes (9.6%) harbored the RPW8 domain. Additionally, 87 genes (63.5%) lacked an N-terminal domain. Most NLR genes in *A. officinalis* and its closely related species contain long coding sequences (CDS), while a minority are formed by the splicing of short CDSs.

### Conserved motif analysis of NLRs

**Figure 3:**
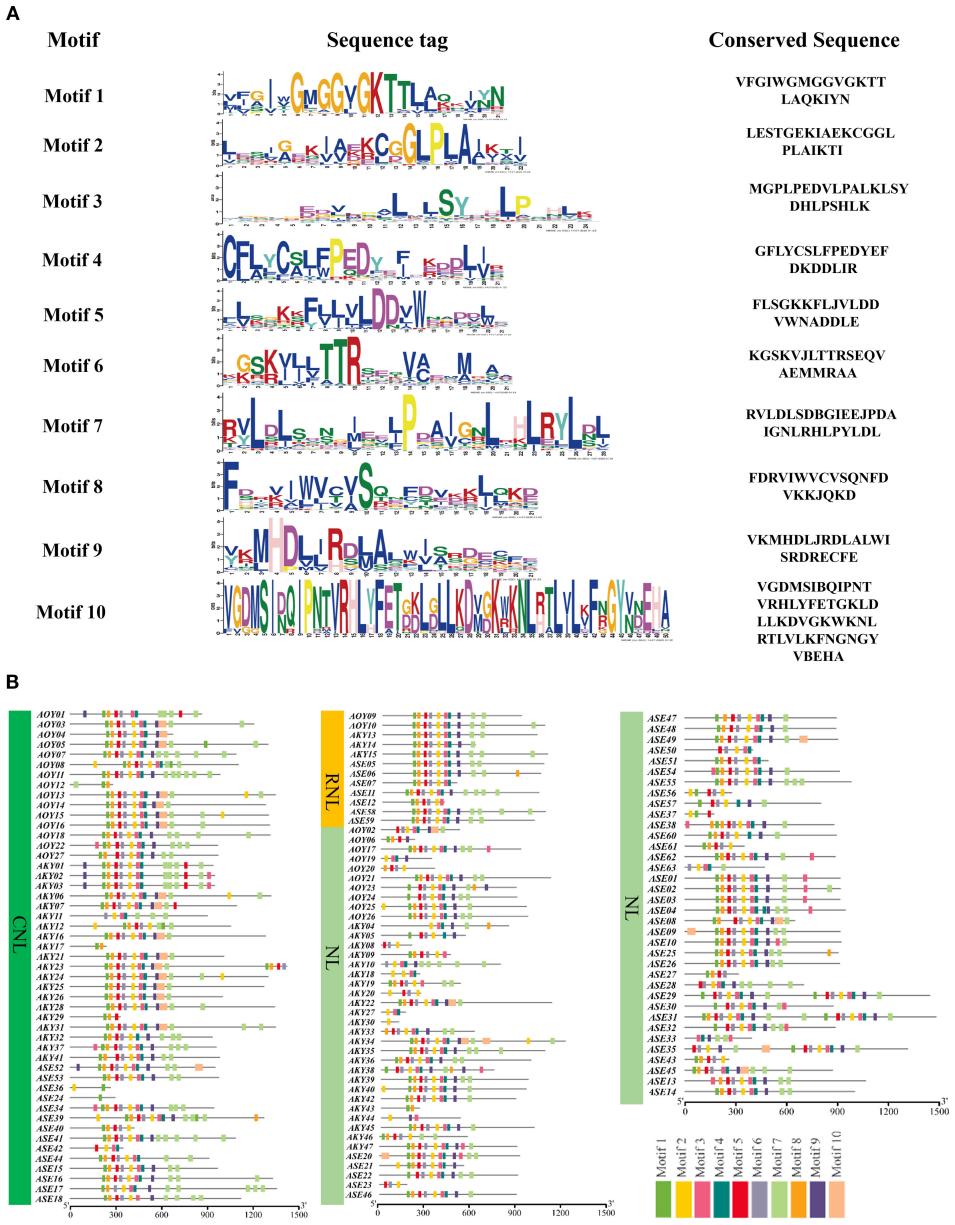
The conserved motifs of NLR gene family members **(A)** and the distribution of these conserved motifs **(B)**.

To elucidate the structural characteristics of NLR proteins, we used MEME (https://meme-suite.org/meme/tools/tomtom) to predict conserved motifs in the amino acid sequences of candidate NLR genes from *A. officinalis* and its closely related species. A total of 10 conserved motifs were identified (**Figure 3A**). Visualization results revealed that three types of NLR proteins (CNL, RNL, and NL) have a relatively consistent variety of motifs within their respective types, indicating that these motifs have conserved functions within each type. The variety of motifs ranges from 2 to 10, which likely reflects the functional diversity of these types of proteins. Motif 7, located at the N-terminus, was present in all CNL and RNL proteins, suggesting structural similarity in their N-terminal domains. However, only a subset of NL-type members retained Motif 7, possibly due to incomplete loss of the N-terminal domain during evolution (**Figure 3B**).

### Promoter cis-element analysis of NLRs in *A. officinalis*, *A. kiusianus*, and *A. setaceus*

The cis-elements in the promoter sequences of NLR genes were predicted using PLANTCARE database. The results showed that five kinds of the disease resistance-related cis-acting elements identified in the three species, which indicated that NLR might participate in stress triggered signaling pathways. The elements contained two kinds of plant-hormone-related elements, MeJA-responsive elements (TGACG-motif and CGTCA-motif) and SA-responsive element (TCA-element), among them, the MeJA-responsive cis-elements were the most abundant across all three NLR classes. On the other hand, other important cis-elements also existed in these promoters, responsive to biotic and abiotic stresses, such as defense/stress-responsive element (TC-rich repeats), drought-inducible MYB binding site (MBS), flavonoid biosynthesis-related MYB binding site (MBSI). Among the total disease resistance-related cis-acting elements, plant hormone elements accounted for 80.8%, stress response elements accounted for 5.5%. However, no significant differences in the number and distribution of promoter elements between different subfamilies were found (**Supplementary Figure S2**).

### Phylogenetic analysis of NLRs in *A. officinalis*, *A. kiusianus*, and *A. setaceus*

**Figure 4:**
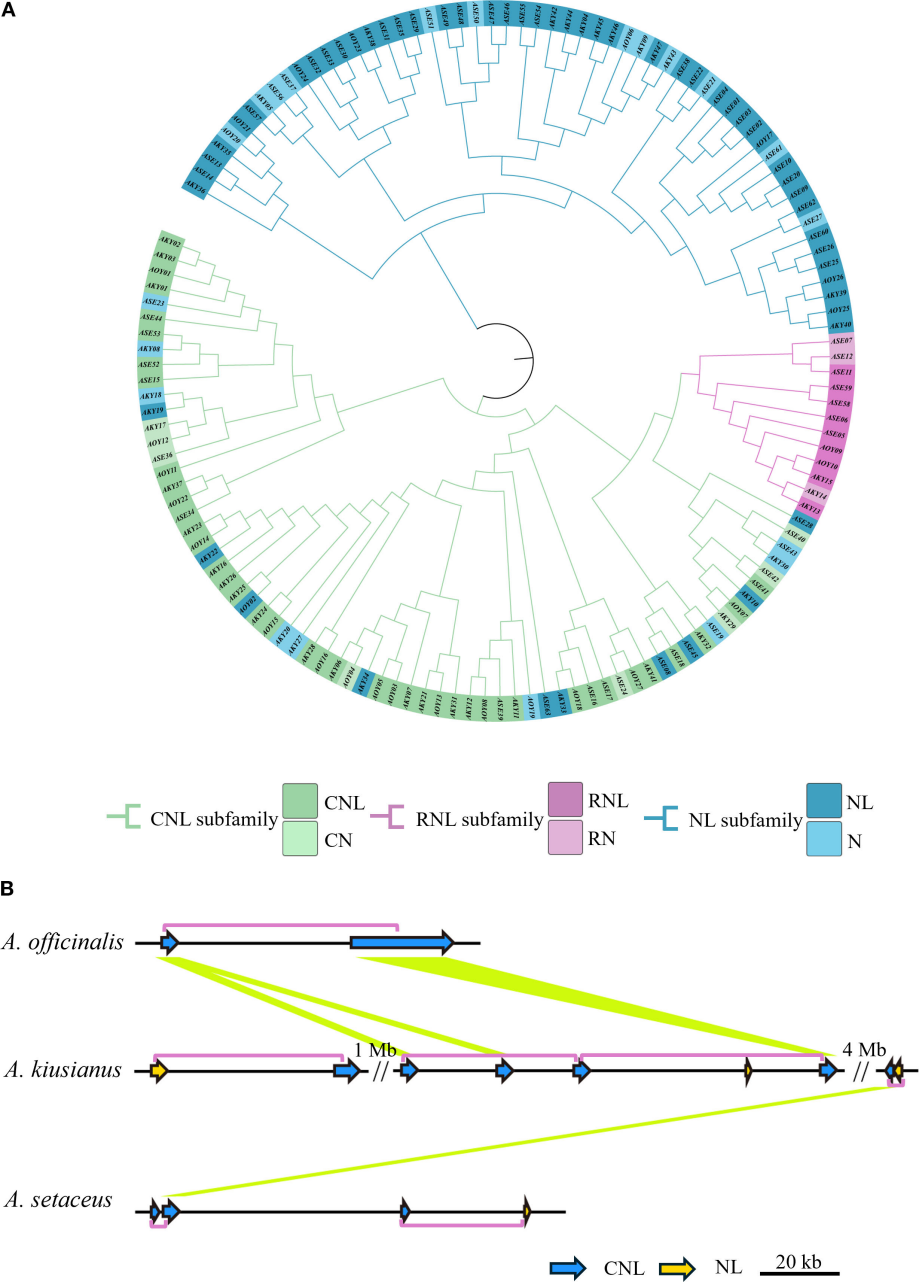
Diversity of NLR genes in three species. **(A)** Phylogenetic analysis of NLR genes from *A*. *officinalis*, *A*. *kiusianus*, and *A*. *setaceus*. **(B)** High prevalence of head-to-tail configured pairs of R genes within an orthologous region across 3 species of *Asparagus*. This complex cluster on chromosome 6 consists of genes from the “CNL” and “NL” families. Purple boxes outline the coupled gene pairs, and yellow lines connect homologous genes (BLASTP ≥ 80%, coverage ≥ 80%). Region shown corresponds to 6: 14, 003, 822-14, 080, 510 on the *A*. *officinalis*.

To clarify the evolutionary relationships of the NLRs in three species, we used IQ-TREE (v. 2.2.0) to construct a maximum likelihood (ML) evolutionary tree based on the Nucleotide binding domain (NB-ARC). The phylogenetic tree divided the NLR proteins into two distinct groups (**Figure 4A**). Based on subfamily identification, it is evident that a subset of NL-type genes occupies distinct branches, with the majority classified as NL-type and a minority as N-type. Approximately 45.9% of the genes belong to the CNL clade, which encompasses diverse NLR genes, suggesting potential tandem duplication events and loss of N-terminal domains during evolution. Notably, some CNL-type genes exhibit species-specific clustering patterns. For instance, genes derived from *A. officinalis* and *A. kiusianus* form separate monophyletic groups within certain branches, indicating independent expansion histories within their respective genomes. The CNL clade further includes RNL (RPW8-NLR-like) genes, which are present in limited numbers and form a relatively independent subclade in the phylogenetic tree. This observation aligns with established reports that RNL represents a distinct subclade of CNLs (Jubic et al., 2019), demonstrating its high evolutionary conservation within the asparagus genus. Additionally, 19 NL-type genes are distributed within the CNL clade. To investigate the underlying reasons, we constructed a phylogenetic tree using the sequences of the LRR domain, and some NL and CNL genes still clustered together (**Supplementary Figure S3**). This indicates that their core modules are homologous. The NL genes that cluster with CNL in the phylogenetic tree evolved from ancestral CNL genes through the loss of the N-terminal CC domain. Essentially, they represent a “degenerate” form of CNL. However, due to the high homology of their core NB-ARC and LRR domains with ancestral CNL, they cluster closely in the phylogenetic tree. Convergent evolution and long-branch attraction may also be contributing factors.

Recent studies have revealed that disease resistance in plants can depend on the cooperative function of two adjacent NLR genes (Okuyama et al., 2011; Cesari et al., 2013). To assess the prevalence of this genetic feature, we identified 81 instances of adjacent NLR gene pairs across the three species: 4 in *A. officinalis*, 32 in *A. kiusianus*, and 45 in *A. setaceus*. Of these, 82.7% are arranged in a head-to-tail orientation (**Supplementary Table S3**). **Figure 4B** illustrates the arrangement of NLR pairs on chromosome 6, where the genes are predominantly organized in a head-to-tail orientation (chi-squared test, *P* = 0.003).

### Natural and artificial selection may have driven the contraction of NLR family members in *A. officinalis*

**Figure 5:**
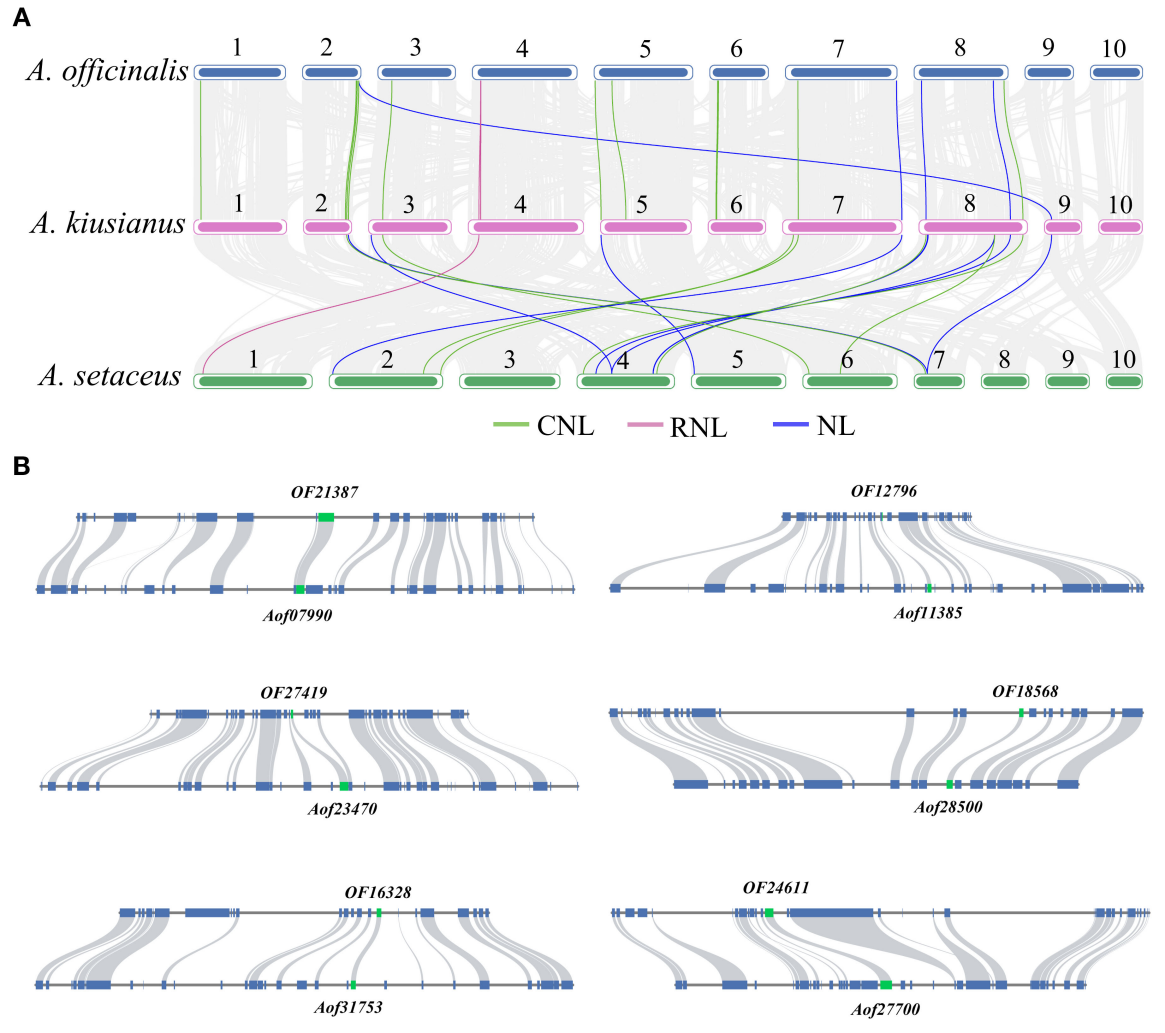
Contraction events of NLR genes in *A*. *officinalis*. **(A)** Collinearity analyses of NLR genes between *A*. *officinalis*, *A. kiusianus*, and *A*. *setaceus*, the blue lines indicate CNL, the purple lines indicate RNL, while the green lines indicate NL. **(B)** Collinearity analyses of orthologous genes (green block) and their neighboring gene pairs (blue block) between *A*. *officinalis* and *A*. *setaceus*.

In order to further clarify the origin and evolution, we constructed the collinearity of the NLR genes in the *A. officinalis, A. kiusianus*, and *A. setaceus* (**Figure 5A**). Syntenic pairs, particularly those of CNL and NL types, are widely distributed across the chromosomes of *A. officinalis*, *A. kiusianus*, and *A. setaceus*, indicating that these genes exhibit conserved genomic arrangements among different species. The CNL type shows abundant highlighted connections, with multiple cross-chromosomal syntenic pairs, suggesting that CNL genes have evolved conservatively in *A. officinalis* and its related species. RNL genes are fewer in number but also display cross-chromosomal conservation, implying that RNL may have diverged earlier in phylogeny. The synteny of NL genes is more scattered, reflecting that NL-type genes have undergone more duplication, translocation, or rearrangement during evolution. Partial CNL and NL genes are missing in the syntenic blocks of *A. officinalis*, suggesting possible gene loss events.

To investigate the duplication events of NLR genes in *A. officinalis* and *A. setaceus*, we used the MCScanX tool to categorize their duplication types. The results showed that no NLR gene pairs attributable to recent whole-genome duplication (WGD) events were detected in *A. officinalis*, nor were any typical high-density NLR tandem clusters identified. In contrast, in the related species *A. setaceus*, we found two NLR gene pairs that likely originated from an ancient WGD event (**Supplementary Figure S4**).

Our findings revealed a distinctive contraction of NLRs in *A. officinalis*. To further identify which NLR genes in *A. officinalis* were retained from evolutionarily distant *A. setaceus*, we selected shared NLR gene pairs between *A. officinalis*, and *A. setaceus* for synteny analysis of their flanking 10–20 genes. The results demonstrated that only 16 NLR genes resides within a homologous block due to strong conservation of its neighboring genes (**Figure 5B**). We used the formula T = Ks/2λ to evaluate approximate dates of 16 duplicated genes (DEs). The dates of shared DEs of NLR genes varied from 7.17 to 30.31 million years ago (Mya) in *A. officinalis* and *A. setaceus* (**Supplementary Table S4**). Then we analyzed the Ka, Ks, and Ka/Ks values of the above-mentioned collinear gene pairs. We found that the Ka/Ks ratios of most collinear gene pairs were less than 1 (**Supplementary Figure S5**), implying these NLRs underwent negative selection in the process of *A. officinalis* evolution.

### Fungal resistance and *A. officinalis* retained NLR expression pattern in response to *Phomopsis asparagi*

**Figure 6:**
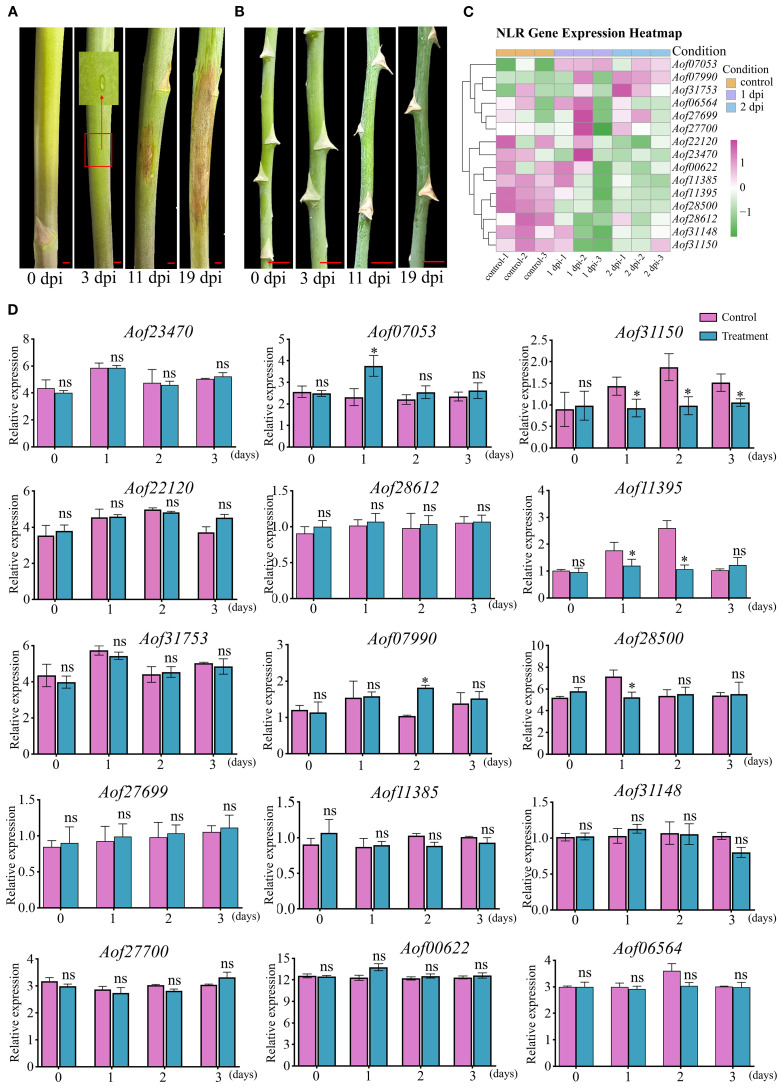
Stem blight resistance of *A*. *officinalis* and *A. setaceus* and expression analysis of asparagus NLR genes at different infection stages. **(A)**. *P. asparagi* infection *A*. *officinalis*, **(B)**. *P. asparagi* infection *A*. *setaceus*, **(C)**. Transcriptional profile analysis *A*. *officinalis* retained NLR during infection plants 1 and 2 dpi with *P. asparagi* and control (0 dpi). Different colors represent the relative expression levels of genes (mean of n = 3 biological replicates), the scale bar on the right was marked according to the Log_2_ (foldchange), and scaled from −1 (green, down-regulated) to +1 (purple, up-regulated); white indicates no change. **(D)**. qRT-PCR validation of fifteen NLR genes. Bars depict mean relative expression ± SE (n = 3 biological replicates). Asterisks denote significant difference. (Tukey’s HSD, *P* ≤ 0.01). Scale bar in inset: 5 mm.

To assess the resistance of *A. officinalis* and *A. setaceus* to pathogenic fungi, we performed artificial inoculation with *P. asparagi* and monitored disease progression systematically. At 3 dpi, necrosis appeared in the centers of lesions on *A. officinalis*; by 11 dpi, most lesions had coalesced; and by 19 dpi, visible stem wilting was evident (**Figure 6A**). In contrast, *A. setaceus* exhibited no conspicuous symptoms at the inoculation site up to 19 dpi (**Figure 6B**). In the transcriptome data of *A. officinalis* treated with *P. asparagi*, 15 retained NLR genes showed expression: 1 gene (*Aof07053*) was up-regulated at 1 dpi, and another (*Aof07990*) at 2 dpi; three genes (*Aof28500, Aof31150, Aof11395*) was down-regulated at 1 dpi, and two (*Aof31150, Aof11395*) at 2 dpi; while the remaining genes showed no significant differences between the treatment and control groups (**Figure 6C**). To assess the expression dynamics of retained NLR genes in response to *P. asparagi* infection, we performed a time-course RT-qPCR analysis at 0, 1, 2, and 3 dpi. Upon pathogen challenge, the transcript levels of the 15 NLR genes were monitored: 11 genes showed no significant difference compared with the mock controls; 3 genes were down-regulated at 1 dpi; 2 genes were down-regulated at 2 dpi; 1 genes were down-regulated at 3 dpi and only 1 genes were up-regulated at 1 dpi, and another gene was up-regulated at 2 dpi (**Figure 6D**). The expression data of these genes revealed patterns consistent with the transcriptome data.

## Discussion

In this study, we first systematically characterized the abundance, classification and characterization of NLR genes in *A. officinalis* and its related species. Most NLR genes are distributed in distinct clusters along the chromosomes, with only a few presents as singleton loci. The identified NLRs were classified into three subfamilies, consistent with previous reports (Guo et al., 2025). In *Oryza*, NLR genes are positionally clustered, with homogeneous pairs arranged in either head-to-tail or head-to-head orientation (Stein et al., 2018). Our analyses reveal analogous head-to-tail pairs in three species suggesting that this evolutionary strategy is conserved across different monocot lineages and represents an important mechanism for forming complex disease-resistance systems. CNL, RNL and NL proteins were simultaneously localized to both the nucleus and the cytoplasm. Pathogen recognition and resistance responses occur in the cytoplasm (Heidrich et al., 2011; Qi and Innes, 2013), indicating that these NLR genes may contribute to pathogen resistance. Most plant immune responses are accompanied by the release of phytohormones (Liang and Zhou, 2018). Cis-element analysis of promoters from *A. officinalis* and its close relatives revealed a significant enrichment of hormone-responsive elements related to SA, ABA and MeJA, it indicated that the *A. officinalis* and its related species NLR family of genes may be participating in the regulation of phytohormone response.

Although comparative analysis shows that NLR genes were very comparable among these three species in terms of number, chromosomal distribution and domain, but the total number of NLR genes showed a significant increasing trend from the cultivated species (*A. officinalis*) to the wild species (*A. kiusianus*) and further to *A. setaceus*. The question of whether resistant cultivars possess more NLRs than susceptible ones remain debated (Jupe et al., 2012; Thatcher et al., 2023). The disease-resistant variety *A. kiusianus* possesses a significantly greater total number of NLR genes compared to the susceptible cultivar *A. officinalis*, suggesting that a higher number of NLRs may contribute to disease resistance in wild species asparagus.

Our findings revealed a distinctive contraction of the NLR gene family in *A. officinalis*, NLR contraction in cultivated asparagus is consistent with a broader trend observed across monocots. In cultivated barley, a recent pan-NLRome study reported a 12-18% reduction in NLR counts relative to wild progenitors; the lost NLRs are primarily concentrated in chromosomal regions associated with yield and quality selection during domestication (Feng et al., 2025). There are fewer NLR genes in maize than in other *Poaceae* (Hufford et al., 2021). In cultivated rice, during high-yield and quality breeding, linkage drag has caused the co-elimination of several major blast-resistance R genes (e.g., Piz-t and Pigm) from breeding populations, resulting in a widespread absence of these resistance loci in elite high-yielding lines (Xiao et al., 2021). Whereas WGD and TD have been major drivers of NLR gene family expansions in various plant lineages, such as *Oryza* and *Arabidopsis* (Stein et al., 2018; Van de Weyer et al., 2019), our analyses found no evidence for recent WGD events or large-scale TD contributing to NLR proliferation in *A. officinalis*. Instead, the expansion of the NLR family in this species appears to rely primarily on alternative mechanisms, such as dispersed duplications or transposon-mediated replication. Furthermore, the lack of retained NLR copies derived from WGD or TD suggests that *A. officinalis* may have undergone strong selective sweeps or persistent purifying selection, effectively eliminated redundant gene duplicates while maintained a ‘lean’ but highly effective immune receptor repertoire. This evolutionary pattern may reflect an adaptation to specific ecological pressures or pathogen environments. Balancing growth and defense against pests and pathogens are critical for plants to maintain high productivity. Insufficient defense responses result in severe yield losses, but excessive constitutive or inducible defense responses inhibit plant growth and development, also decreasing yields (Sun et al., 2025). The need to balance growth and defense has likely been a driving force during the evolution and domestication of plants (Zhang et al., 2020). For example, during the domestication of cultivated soybean (*Glycine max*) from its wild progenitor (*Glycine soja*), human-mediated selection led to the development of improved cultivars characterized by enhanced growth capacity and larger seeds, as well as notable advancements in yield and quality. Nevertheless, these advantages were accompanied by a reduced ability to endure environmental stresses (Zhou et al., 2015). NLR gene family contraction in *A. officinalis*, which might be related to the artificial domestication and environmental adaptation of *A. officinalis*, potentially reflecting a trade-off between growth and defense.

To validate the hypotheses derived from these genomic studies, we further identified NLR orthologous genes in *A. officinalis* and *A. setaceus*, the flanking genes of orthologous gene pairs exhibited strong synteny. Purifying selection plays a critical role in eliminating disadvantageous mutations, thereby maintaining the stability and functionality of essential genes (Van de Weyer et al., 2019), this was reflected in our study, where the Ka/Ks ratio of duplicated NLR gene pairs was less than 1 indicating the retained gene functions are highly conserved and may be essential for the basic survival and reproduction of organisms. We also investigated the physiological functions of the retained NLR genes in *A. officinalis*. After fungal infection, *A. officinalis* showed distinct stem blight symptoms on the stem surface from 3 dpi, while *A. setaceus* exhibited no symptoms even at 19 dpi. The expression levels of most NLR genes in *A. officinalis* either remained unchanged or were downregulated, suggesting that these genes have may not be effectively mobilized during the compatible interaction with *P. asparagi*. These findings provide valuable information that could aid future studies of the physiological mechanisms of NLR in *A. officinalis* disease resistance. Future work will aim to establish a stable genetic transformation system for *A. officinalis* to directly test the necessity and sufficiency of specific NLR genes in disease resistance and to unequivocally determine the impact of domestication on their signaling functions.

In conclusion, this study represents the first comprehensive elucidation of the distribution patterns and evolutionary characteristics of NLR genes in *A. officinalis* and its closely related species. The heightened susceptibility of *A. officinalis* to pathogens may be attributed to the contraction of its NLR gene repertoire and the ineffectiveness of the retained NLR genes in mounting robust immune responses. The reduction of NLR genes is likely associated with the prioritization of quality and yield enhancement as primary breeding objectives during artificial selection in *A. officinalis*. This study offers valuable insights for breeding programs focused on enhancing disease resistance in the important vegetable crop garden asparagus.

## Data Availability

The datasets presented in this study can be found in online repositories. The names of the repository/repositories and accession number(s) can be found in the article/**Supplementary Material**.

## References

[B1] Abdelrahman M. Suzumura N. Mitoma M. Matsuo S. Ikeuchi T. Mori M.. (2017). Comparative *de novo* transcriptome profiles in *Asparagus officinalis* and *A. Kiusianus* during the early stage of *Phomopsis asparagi* infection. Sci. Rep. 7, 2608. doi: 10.1038/s41598-017-02566-7, PMID: 28572584 PMC5453997

[B2] Bailey T. L. Johnson J. Grant C. E. Noble W. S. (2015). The MEME suite. Nucleic. Acids Res. 43, W39–W49. doi: 10.1093/nar/gkv416, PMID: 25953851 PMC4489269

[B3] Cesari S. Thilliez G. Ribot C. Chalvon V. Michel C. Jauneau A.. (2013). The rice resistance protein pair RGA4/RGA5 recognizes the *Magnaporthe oryzae* effectors AVR-Pia and AVR1-CO39 by direct binding. Plant Cell 25, 1463–1481. doi: 10.1105/tpc.112.107201, PMID: 23548743 PMC3663280

[B4] Chen C. Chen H. Zhang Y. Thomas H. R. Frank M. H. He Y.. (2020). TBtools: an integrative toolkit developed for interactive analyses of big biological data. Mol. Plant 13, 1194–1202. doi: 10.1016/j.molp.2020.06.009, PMID: 32585190

[B5] Eitas T. K. Dangl J. L. (2010). NB-LRR proteins: pairs, pieces, perception, partners, and pathways. Curr. Opin. Plant Biol. 13, 472–477. doi: 10.1016/j.pbi.2010.04.007, PMID: 20483655 PMC2910844

[B6] Emms D. M. Kelly S. (2019). OrthoFinder: phylogenetic orthology inference for comparative genomics. Genome Biol. 20, 238. doi: 10.1186/s13059-019-1832-y, PMID: 31727128 PMC6857279

[B7] Feng J. W. Pidon H. Cuacos M. Lux T. Himmelbach A. Haghi R.. (2025). A haplotype-resolved pangenome of the barley wild relative *Hordeum bulbosum* . Nature. 645, 429–438. doi: 10.21203/rs.3.rs-3916840/v1, PMID: 40634612 PMC12422954

[B8] Guo B. Zhang Y. Liu Z. Li X. Yu Z. Ping B.. (2025). Deciphering plant NLR genomic evolution: synteny-informed classification unveils insights into TNL gene loss. Mol. Biol. Evol. 42, msaf015. doi: 10.1093/molbev/msaf015, PMID: 39835721 PMC11789945

[B9] Harkess A. Zhou J. Xu C. Bowers J. E. van der Hulst R. Ayyampalayam S.. (2017). The asparagus genome sheds light on the origin and evolution of a young y chromosome. Nat. Commun. 8, 1279. doi: 10.1038/s41467-017-01064-8, PMID: 29093472 PMC5665984

[B10] Heidrich K. Wirthmueller L. Tasset C. Pouzet C. Deslandes L. Parker J. E. (2011). *Arabidopsis* EDS1 connects pathogen effector recognition to cell compartment-specific immune responses. Science 334, 1401–1404. doi: 10.1126/science.1211641, PMID: 22158818

[B11] Horton P. Park K. Obayashi T. Fujita N. Harada H. Adams-Collier C. J.. (2007). WoLF PSORT: protein localization predictor. Nucleic. Acids Res. 35, W585–W587. doi: 10.1093/nar/gkm259, PMID: 17517783 PMC1933216

[B12] Hu B. Jin J. Guo A. Zhang H. Luo J. Gao G. (2015). GSDS 2.0: an upgraded gene feature visualization server. Bioinformatics 31, 1296–1297. doi: 10.1093/bioinformatics/btu817, PMID: 25504850 PMC4393523

[B13] Hufford M. B. Seetharam A. S. Woodhouse M. R. Chougule K. M. Ou S. Liu J.. (2021). *De novo* assembly, annotation, and comparative analysis of 26 diverse maize genomes. Science 373, 655–662. doi: 10.1126/science.abg5289, PMID: 34353948 PMC8733867

[B14] Ito T. Konno I. Kubota S. Ochiai T. Sonoda T. Hayashi Y.. (2011). Production and characterization of interspecific hybrids between *Asparagus kiusianus* Makino and *A. officinalis* L. Euphytica 182, 285–294. doi: 10.1007/s10681-011-0508-9

[B15] Jones P. Binns D. Chang H. Fraser M. Li W. McAnulla C.. (2014). InterProScan 5: genome-scale protein function classification. Bioinformatics 30, 1236–1240. doi: 10.1093/bioinformatics/btu031, PMID: 24451626 PMC3998142

[B16] Jubic L. M. Saile S. Furzer O. J. El Kasmi F. Dangl J. L. (2019). Help wanted: helper NLRs and plant immune responses. Curr. Opin. Plant Biol. 50, 82–94. doi: 10.1016/j.pbi.2019.03.013, PMID: 31063902

[B17] Jupe F. Pritchard L. Etherington G. J. Mackenzie K. Cock P. J. A. Wright F.. (2012). Identification and localisation of the NB-LRR gene family within the potato genome. BMC Genomics 13, 75. doi: 10.1186/1471-2164-13-75, PMID: 22336098 PMC3297505

[B18] Lee H. Yeom S. (2015). Plant NB-LRR proteins: tightly regulated sensors in a complex manner. Brief. Funct. Genomics 14, 233–242. doi: 10.1093/bfgp/elv012, PMID: 25825425

[B19] Lescot M. Dehais P. Thijs G. Marchal K. Moreau Y. Van de Peer Y.. (2002). PlantCARE, a database of plant cis-acting regulatory elements and a portal to tools for in silico analysis of promoter sequences. Nucleic. Acids Res. 30, 325–327. doi: 10.1093/nar/30.1.325, PMID: 11752327 PMC99092

[B20] Li S. Wang J. Dong R. Zhu H. Lan L. Zhang Y.. (2020). Chromosome-level genome assembly, annotation and evolutionary analysis of the ornamental plant *Asparagus setaceus* . Hortic. Res. 7, 48. doi: 10.1038/s41438-020-0271-y, PMID: 32257234 PMC7109074

[B21] Liang X. Zhou J. (2018). Receptor-like cytoplasmic kinases: central players in plant receptor kinase-mediated signaling. Annu. Rev. Plant Biol. 69, 267–299. doi: 10.1146/annurev-arplant-042817-040540, PMID: 29719165

[B22] Liu Y. Zeng Z. Zhang Y. Li Q. Jiang X. Jiang Z.. (2021). An angiosperm NLR atlas reveals that NLR gene reduction is associated with ecological specialization and signal transduction component deletion. Mol. Plant 14, 2015–2031. doi: 10.1016/j.molp.2021.08.001, PMID: 34364002

[B23] Love M. I. Huber W. Anders S. (2014). Moderated estimation of fold change and dispersion for RNA-seq data with DESeq2. Genome Biol. 15, 550. doi: 10.1186/s13059-014-0550-8, PMID: 25516281 PMC4302049

[B24] Mistry J. Chuguransky S. Williams L. Qureshi M. Salazar G. A. Sonnhammer E. L. L.. (2021). Pfam: the protein families database in 2021. Nucleic. Acids Res. 49, D412–D419. doi: 10.1093/nar/gkaa913, PMID: 33125078 PMC7779014

[B25] Nepal M. P. Andersen E. J. Neupane S. Benson B. V. (2017). Comparative genomics of non-TNL disease resistance genes from six plant species. Genes 8, 249. doi: 10.3390/genes8100249, PMID: 28973974 PMC5664099

[B26] Neupane S. Andersen E. J. Neupane A. Nepal M. P. (2018). Genome-wide identification of NBS-encoding resistance genes in sunflower (*Helianthus annuus* L.). Genes 9, 384. doi: 10.3390/genes9080384, PMID: 30061549 PMC6115920

[B27] Okuyama Y. Kanzaki H. Abe A. Yoshida K. Tamiru M. Saitoh H.. (2011). A multifaceted genomics approach allows the isolation of the rice Pia-blast resistance gene consisting of two adjacent NBS-LRR protein genes. Plant J. 66, 467–479. doi: 10.1111/j.1365-313X.2011.04502.x, PMID: 21251109

[B28] Patro R. Duggal G. Love M. I. Irizarry R. A. Kingsford C. (2017). Salmon provides fast and bias-aware quantification of transcript expression. Nat. Methods 14, 417–419. doi: 10.1038/nmeth.4197, PMID: 28263959 PMC5600148

[B29] Qi D. Innes R. W. (2013). Recent advances in plant NLR structure, function, localization, and signaling. Front. Immunol. 4. doi: 10.3389/fimmu.2013.00348, PMID: 24155748 PMC3801107

[B30] Seo E. Kim S. Yeom S. Choi D. (2016). Genome-wide comparative analyses reveal the dynamic evolution of nucleotide-binding leucine-rich repeat gene family among solanaceae plants. Front. Plant Sci. 7. doi: 10.3389/fpls.2016.01205, PMID: 27559340 PMC4978739

[B31] Shao Z. Xue J. Wu P. Zhang Y. Wu Y. Hang Y.. (2016). Large-scale analyses of angiosperm nucleotide-binding site-leucine-rich repeat genes reveal three anciently diverged classes with distinct evolutionary patterns. Plant Physiol. 170, 2095–2109. doi: 10.1104/pp.15.01487, PMID: 26839128 PMC4825152

[B32] Shirasawa K. Ueta S. Murakami K. Abdelrahman M. Kanno A. Isobe S. (2022). Chromosome-scale haplotype-phased genome assemblies of the male and female lines of wild asparagus (*Asparagus kiusianus*), a dioecious plant species. DNA Res. 29, dsac002. doi: 10.1093/dnares/dsac002, PMID: 35040911 PMC8826022

[B33] Sievers F. Higgins D. G. (2018). Clustal omega for making accurate alignments of many protein sequences. Protein. Sci. 27, 135–145. doi: 10.1002/pro.3290, PMID: 28884485 PMC5734385

[B34] Steele J. F. C. Hughes R. K. Banfield M. J. (2019). Structural and biochemical studies of an NB-ARC domain from a plant NLR immune receptor. PLoS One 14, e0221226. doi: 10.1371/journal.pone.0221226, PMID: 31461469 PMC6713354

[B35] Stein J. C. Yu Y. Copetti D. Zwickl D. J. Zhang L. Zhang C.. (2018). Genomes of 13 domesticated and wild rice relatives highlight genetic conservation, turnover and innovation across the genus *Oryza* . Nat. Genet. 50, 285–296. doi: 10.1038/s41588-018-0040-0, PMID: 29358651

[B36] Sun L. Li Y. Li X. Ruan X. Zhao Y. Wen R.. (2024). Cytological and ultrastructural investigation of pathogen infection pathway and host responses in asparagus stem infected by *Phomopsis asparagi* . Phytopathol. Res. 6, 32. doi: 10.1186/s42483-024-00252-x

[B37] Sun Z. Zhang K. Peng J. Liu B. Kong F. Sang Q.. (2025). Strategies for balancing growth and defence against biotic stress in legumes. Plant Cell Environ. 48(10). doi: 10.1111/pce.70070, PMID: 40665866

[B38] Tameling W. I. L. Baulcombe D. C. (2007). Physical association of the NB-LRR resistance protein rx with a ran GTPase-activating protein is required for extreme resistance to potato virus x. Plant Cell. 19, 1682–1694. doi: 10.1105/tpc.107.050880, PMID: 17526750 PMC1913736

[B39] Thatcher S. Jung M. Panangipalli G. Fengler K. Sanyal A. Li B.. (2023). The NLRomes of *Zea mays* NAM founder lines and *Zea luxurians* display presence-absence variation, integrated domain diversity, and mobility. Mol. Plant Pathol. 24, 742–757. doi: 10.1111/mpp.13319, PMID: 36929631 PMC10257044

[B40] Van de Weyer A. Monteiro F. Furzer O. J. Nishimura M. T. Cevik V. Witek K.. (2019). A species-wide inventory of NLR genes and alleles in *Arabidopsis thaliana* . Cell 178, 1260–1272. doi: 10.1016/j.cell.2019.07.038, PMID: 31442410 PMC6709784

[B41] van Wersch S. Tian L. Hoy R. Li X. (2020). Plant NLRs: the whistleblowers of plant immunity. Plant Commun. 1, 100016. doi: 10.1016/j.xplc.2019.100016, PMID: 33404540 PMC7747998

[B42] Wang J. Song W. Chai J. (2023). Structure, biochemical function, and signaling mechanism of plant NLRs. Mol. Plant 16, 75–95. doi: 10.1016/j.molp.2022.11.011, PMID: 36415130

[B43] Wang Y. Tang H. DeBarry J. D. Tan X. Li J. Wang X.. (2012). MCScanX: a toolkit for detection and evolutionary analysis of gene synteny and collinearity. Nucleic Acids Res. 40, e49. doi: 10.1093/nar/gkr1293, PMID: 22217600 PMC3326336

[B44] Wang D. Zhang Y. Zhang Z. Zhu J. Yu J. (2010). KaKs_calculator 2.0: a toolkit incorporating gamma-series methods and sliding window strategies. Genom. Proteom. Bioinf. 8, 77–80. doi: 10.1016/S1672-0229(10)60008-3, PMID: 20451164 PMC5054116

[B45] Xiao N. Pan C. Li Y. Wu Y. Cai Y. Lu Y.. (2021). Genomic insight into balancing high yield, good quality, and blast resistance of *japonica* rice. Genome Biol. 22, 283. doi: 10.1186/s13059-021-02488-8, PMID: 34615543 PMC8493723

[B46] Xue J. Zhao T. Liu Y. Liu Y. Zhang Y. Zhang G.. (2019). Genome- wide analysis of the nucleotide binding site leucine-rich repeat genes of four orchids revealed extremely low numbers of disease resistance genes. Front. Genet. 10. doi: 10.3389/fgene.2019.01286, PMID: 31998358 PMC6960632

[B47] Yang Z. (2007). PAML 4: phylogenetic analysis by maximum likelihood. Mol. Biol. Evol. 24, 1586–1591. doi: 10.1093/molbev/msm088, PMID: 17483113

[B48] Yu J. Wang U. Lin W. Li S. Li H. Zhou J.. (2005). The genomes of *Oryza sativa*: a history of duplications. PLoS Biol. 3, e38. doi: 10.1371/journal.pbio.0030038, PMID: 15685292 PMC546038

[B49] Zhang H. Zhao Y. Zhu J. (2020). Thriving under stress: how plants balance growth and the stress response. Dev. Cell 55, 529–543. doi: 10.1016/j.devcel.2020.10.012, PMID: 33290694

[B50] Zhou Z. Jiang Y. Wang Z. Gou Z. Lyu J. Li W.. (2015). Resequencing 302 wild and cultivated accessions identifies genes related to domestication and improvement in soybean. Nat. Biotechnol. 33, 408–414. doi: 10.1038/nbt.3096, PMID: 25643055

