# Supplementary material for: Comparative analysis of the NLR gene family in the genomes of garden asparagus (Asparagus officinalis) and its wild relatives
Source: Front Plant Sci. 2025 Sep 25;16:1681919. doi: 10.3389/fpls.2025.1681919 (PMC12507765; doi:10.3389/fpls.2025.1681919)
Supplement: Supplementary file 1 [file DataSheet1.docx]

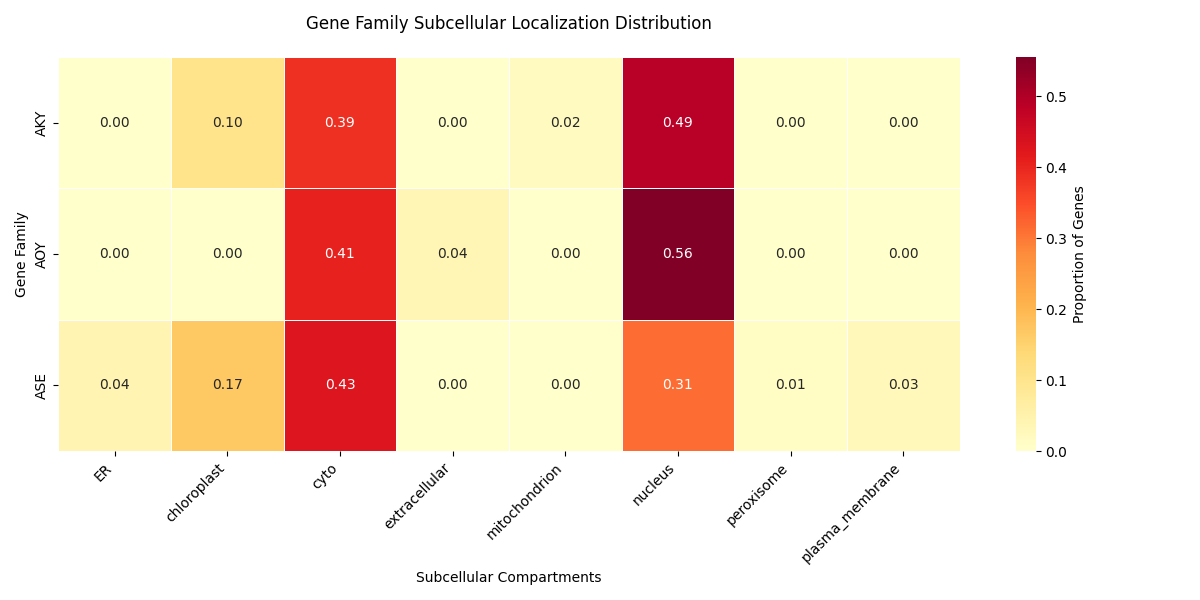


Figure S1. Subcellular localization of NLR gene family members.


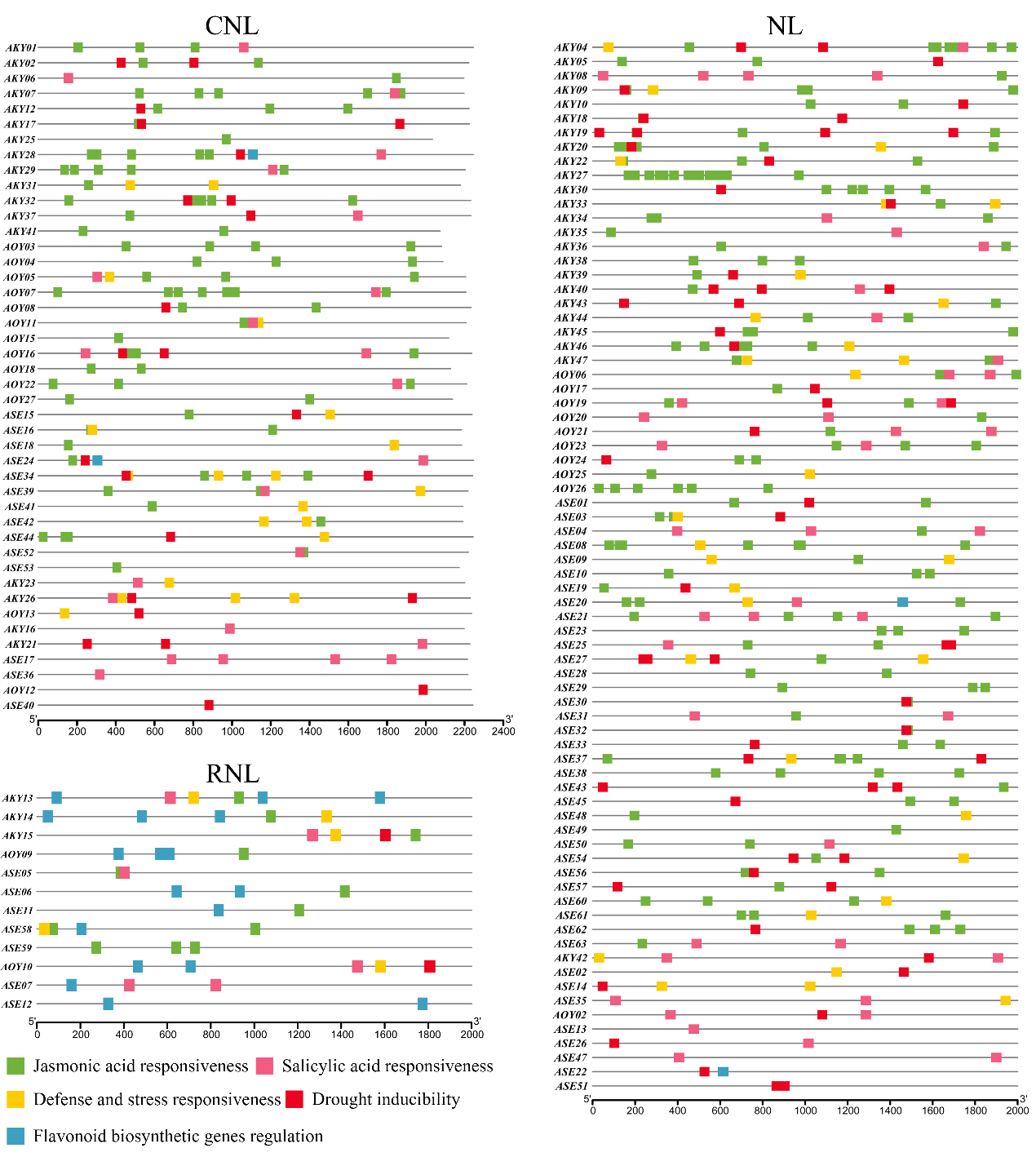


Figure S2. Predicted cis-elements in the promoter regions of NLR genes. Motif names were shown nearby with different colors.


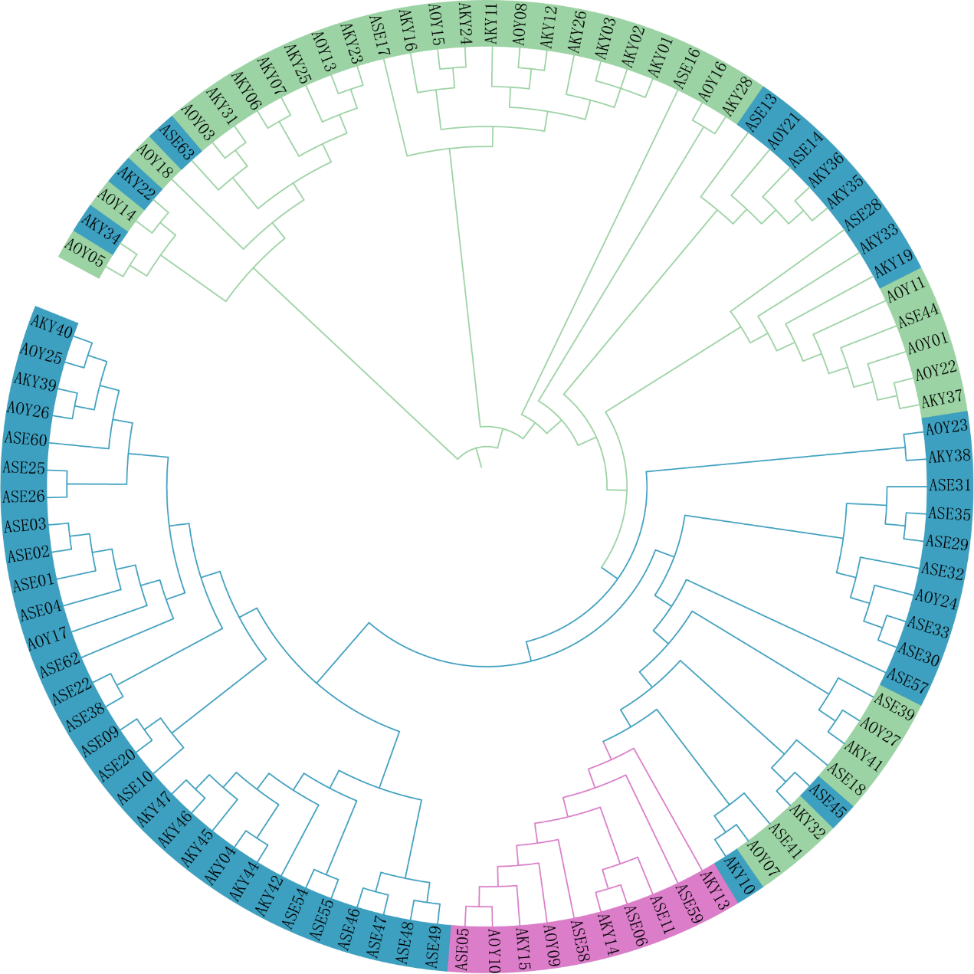


Figure S3. Phylogenetic analysis of NLR genes from *A. officinalis, A. kiusianus,* and *A. setaceus* based on LRR domain.


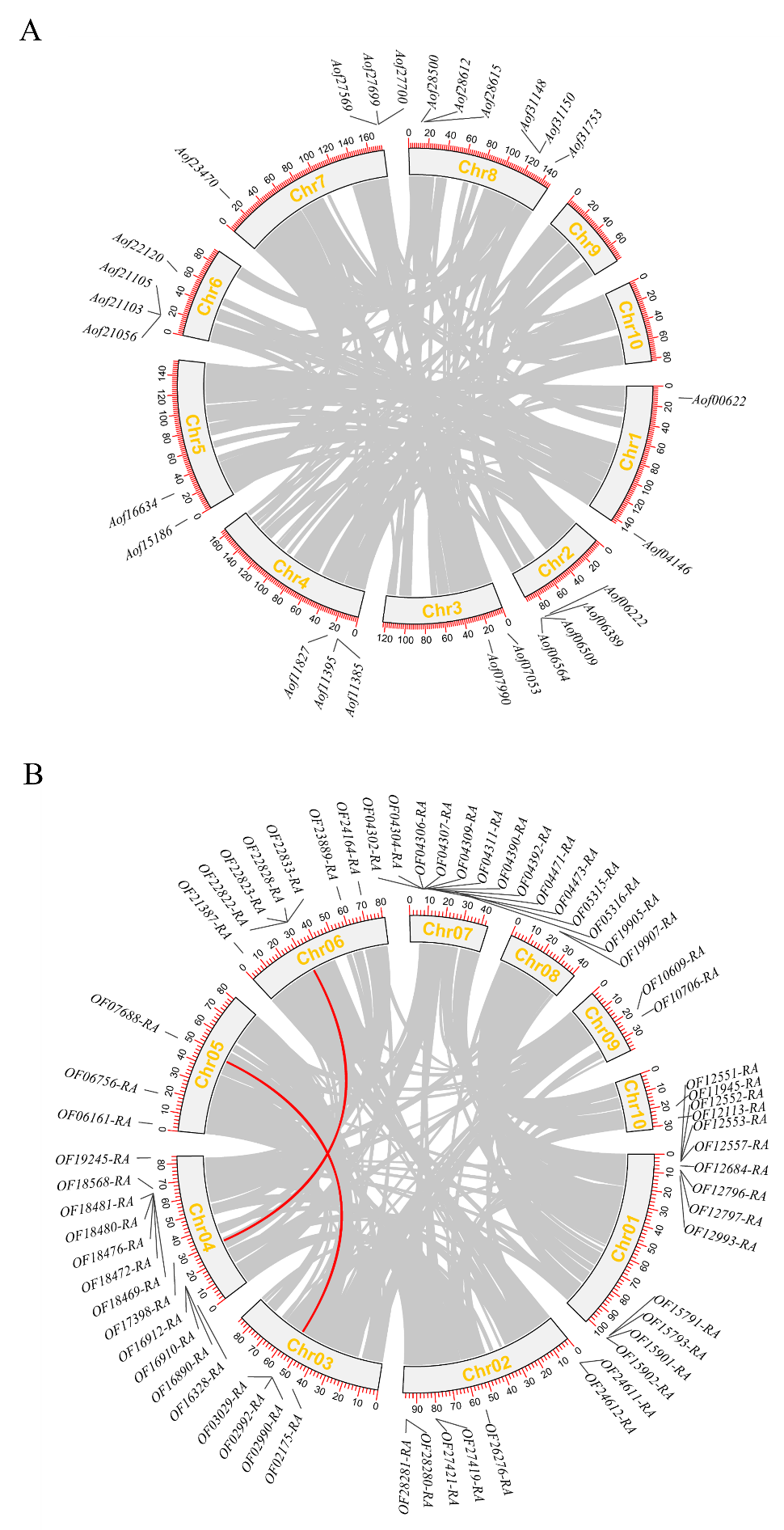


Figure S4. Collinearity blocks containing whole genome duplication gene pairs in (A) *A. officinalis*, and (B) *A. setaceus*, with red lines indicating NLR gene pairs.


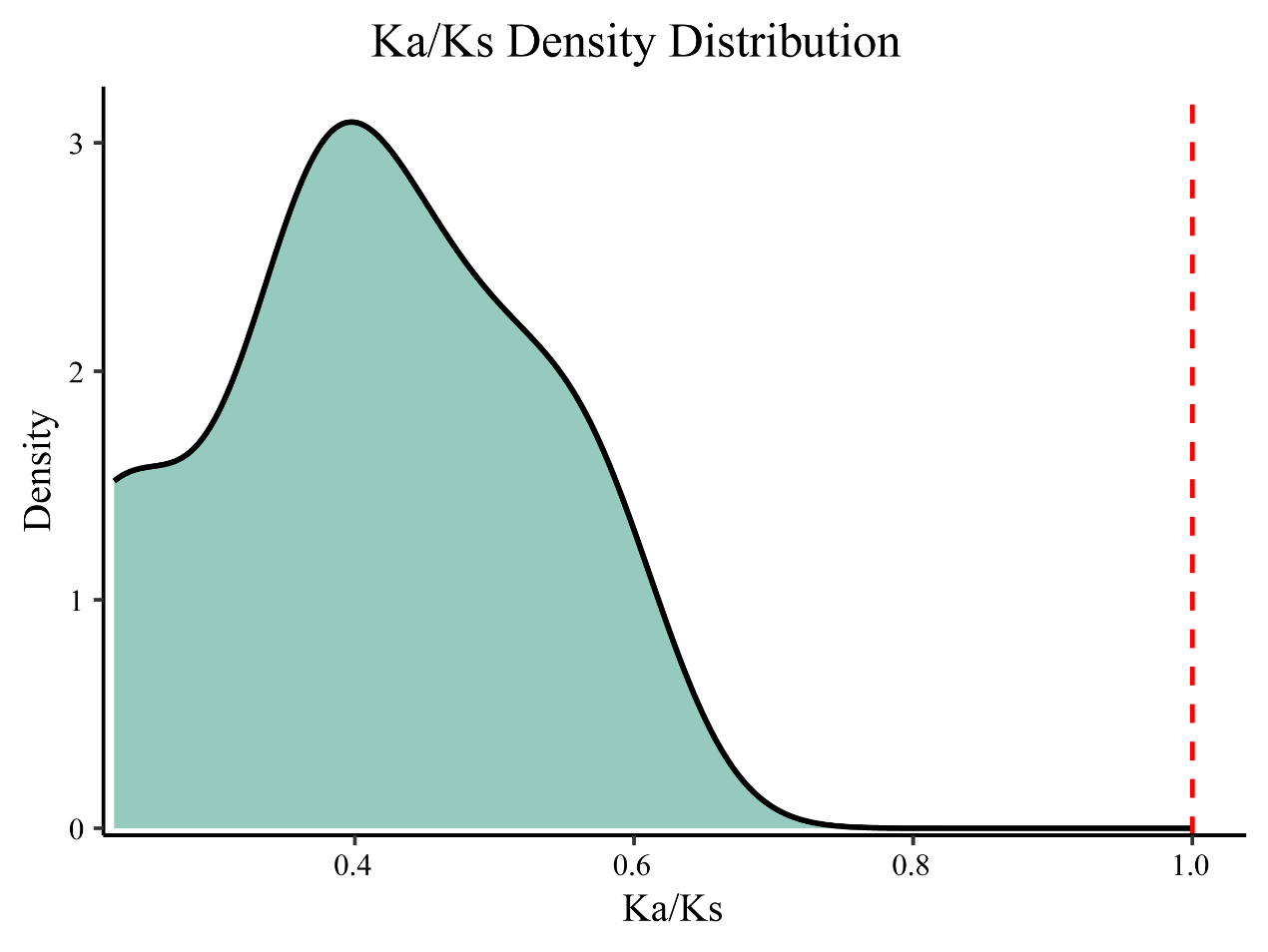


Figure S5. Ka/Ks values of NLR orthologous gene pairs between *A. officinalis*, and *A. setaceus.*

Table S1. Primer pairs used in this study

| **Gene name** | **Forward primer** | **Reverse primer** |
| --- | --- | --- |
| *Aof23470* | AGTGTACGCCCAGTGGAA | TCTGTAGAGCCATATCCCA |
| *Aof31150* | CTTCCCCGCTCTCAGAACTC | GAAGCCTCTTCAGACTCGGG |
| *Aof07990* | CCTACTATCCAGATTGCCC | TGAACCTCGGAGCTCCTTC |
| *Aof28612* | TGAGAGATCTCCGCGAAGCC | GTTTTTCAGCCCTCTGCCTC |
| *Aof22120* | TGCAGAGACCGGATCAGAGA | GTGAAACCCCGATGATGGGT |
| *Aof07053* | AAGCTACCTGCCGATGATGG | AGCCCGTCGCATTTTTCAAC |
| *Aof11385* | ACGACGAAGGAGGACTACCG | TGATTGACTCCGGCAAGCTG |
| *Aof31148* | TAGTCTACCCCGTCTGGAGC | GTATCCTCCCACCACCCTCT |
| *Aof31753* | TGAAGCGCTCATGGTCTCTG | CTGCATTTCTCGGTTGTGGC |
| *Aof28500* | AAGAACTGGGTGAGGGAGGT | TGATTGCCTGCGAACCTGAA |
| *Aof27699* | AAAGCGTGTGGGAGAGTGG | CGCAACTTCCAACCTCAAGC |
| *Aof27700* | GAAGGGGACTCCTTTCGCTC | TTGAGATCGGCGACTGTGTC |
| *Aof00622* | GAATGGCTGCGCTCTTTGTC | TCGGCATTGGAAGAACCCTC |
| *Aof06564* | GAGCTTGCCTGTTGGAGGA | CTCCGTTTTGGAACATCCAAC |
| *Aof11395* | TGCATGTTTATGGCTGCCCA | TCGACGATGTCCAACGAACC |
| *EF1*A | GTGGCAGGGTGGTTCATGAT | TAAGTCTGTTGAGATGCACC |

Table S2. NLR classification and renaming

| **Species** | **Gene ID** | **Classification** | **renaming** |
| --- | --- | --- | --- |
| *A. officinalis* | *Aof00622* | CNL | *AOY01* |
|  | *Aof04146* | NL | *AOY02* |
|  | *Aof06222* | CNL | *AOY03* |
|  | *Aof06389* | CN | *AOY04* |
|  | *Aof06509* | CNL | *AOY05* |
|  | *Aof06564* | N | *AOY06* |
|  | *Aof07053* | CNL | *AOY07* |
|  | *Aof07990* | CNL | *AOY08* |
|  | *Aof11385* | RNL | *AOY09* |
|  | *Aof11395* | RNL | *AOY10* |
|  | *Aof11827* | CNL | *AOY11* |
|  | *Aof15186* | CN | *AOY12* |
|  | *Aof16634* | CNL | *AOY13* |
|  | *Aof21056* | CNL | *AOY14* |
|  | *Aof21103* | CNL | *AOY15* |
|  | *Aof21105* | CNL | *AOY16* |
|  | *Aof22120* | NL | *AOY17* |
|  | *Aof23470* | CNL | *AOY18* |
|  | *Aof27569* | N | *AOY19* |
|  | *Aof27699* | N | *AOY20* |
|  | *Aof27700* | NL | *AOY21* |
|  | *Aof28500* | CNL | *AOY22* |
|  | *Aof28612* | NL | *AOY23* |
|  | *Aof28615* | NL | *AOY24* |
|  | *Aof31148* | NL | *AOY25* |
|  | *Aof31150* | NL | *AOY26* |
|  | *Aof31753* | CNL | *AOY27* |
| *A. kiusianus* | *K1p1ch01g28595* | CNL | *AKY01* |
|  | *K1p1ch01g28596* | CNL | *AKY02* |
|  | *K1p1ch01g28614* | CNL | *AKY03* |
|  | *K1p1ch01g29271* | NL | *AKY04* |
|  | *K1p1ch02g53615* | N | *AKY05* |
|  | *K1p1ch02g55712* | CNL | *AKY06* |
|  | *K1p1ch02g55808* | CNL | *AKY07* |
|  | *K1p1ch02g55941* | N | *AKY08* |
|  | *K1p1ch02g56105* | N | *AKY09* |
|  | *K1p1ch03g00325* | NL | *AKY10* |
| **Continued** |  |  |  |
| **Species** | **Gene ID** | **Classification** | **renaming** |
| *A. kiusianus* | *K1p1ch03g01635* | CNL | *AKY11* |
|  | *K1p1ch03g01636* | CNL | *AKY12* |
|  | *K1p1ch04g21098* | RNL | *AKY13* |
|  | *K1p1ch04g21101* | RN | *AKY14* |
|  | *K1p1ch04g21163* | RNL | *AKY15* |
|  | *K1p1ch04g25723* | CNL | *AKY16* |
|  | *K1p1ch05g06225* | CN | *AKY17* |
|  | *K1p1ch05g06230* | N | *AKY18* |
|  | *K1p1ch05g06232* | NL | *AKY19* |
|  | *K1p1ch05g06675* | N | *AKY20* |
|  | *K1p1ch05g08001* | CNL | *AKY21* |
|  | *K1p1ch06g35722* | NL | *AKY22* |
|  | *K1p1ch06g35725* | CNL | *AKY23* |
|  | *K1p1ch06g35768* | CNL | *AKY24* |
|  | *K1p1ch06g35769* | CNL | *AKY25* |
|  | *K1p1ch06g35771* | CNL | *AKY26* |
|  | *K1p1ch06g35775* | N | *AKY27* |
|  | *K1p1ch06g35778* | CNL | *AKY28* |
|  | *K1p1ch06g35927* | CN | *AKY29* |
|  | *K1p1ch06g35928* | N | *AKY30* |
|  | *K1p1ch06g38186* | CNL | *AKY31* |
|  | *K1p1ch07g39242* | CNL | *AKY32* |
|  | *K1p1ch07g39657* | NL | *AKY33* |
|  | *K1p1ch07g42693* | NL | *AKY34* |
|  | *K1p1ch07g46492* | NL | *AKY35* |
|  | *K1p1ch07g46493* | NL | *AKY36* |
|  | *K1p1ch08g13858* | CNL | *AKY37* |
|  | *K1p1ch08g14048* | NL | *AKY38* |
|  | *K1p1ch08g18532* | NL | *AKY39* |
|  | *K1p1ch08g18536* | NL | *AKY40* |
|  | *K1p1ch08g19491* | CNL | *AKY41* |
|  | *K1p1ch09g57372* | NL | *AKY42* |
|  | *K1p1ch09g57376* | N | *AKY43* |
|  | *K1p1ch09g57377* | NL | *AKY44* |
|  | *K1p1ch09g57378* | NL | *AKY45* |
|  | *K1p1ch09g57379* | NL | *AKY46* |
|  | *K1p1ch09g57386* | NL | *AKY47* |
| **Continued** |  |  |  |
| **Species** | **Gene ID** | **Classification** | **renaming** |
| *A. kiusianus* | *K1p1ch01g28595* | CNL | *AKY01* |
|  | *K1p1ch01g28596* | CNL | *AKY02* |
| *A. setaceus* | *OF12551* | NL | *ASE01* |
|  | *OF12552* | NL | *ASE02* |
|  | *OF12553* | NL | *ASE03* |
|  | *OF12557* | NL | *ASE04* |
|  | *OF12684* | RNL | *ASE05* |
|  | *OF12796* | RNL | *ASE06* |
|  | *OF12797* | RN | *ASE07* |
|  | *OF12993* | NL | *ASE08* |
|  | *OF15791* | NL | *ASE09* |
|  | *OF15793* | NL | *ASE10* |
|  | *OF15901* | RNL | *ASE11* |
|  | *OF15902* | RN | *ASE12* |
|  | *OF24611* | NL | *ASE13* |
|  | *OF24612* | NL | *ASE14* |
|  | *OF26276* | CNL | *ASE15* |
|  | *OF27419* | CNL | *ASE16* |
|  | *OF27421* | CNL | *ASE17* |
|  | *OF28280* | CNL | *ASE18* |
|  | *OF28281* | N | *ASE19* |
|  | *OF02175* | NL | *ASE20* |
|  | *OF02990* | N | *ASE21* |
|  | *OF02992* | NL | *ASE22* |
|  | *OF03029* | N | *ASE23* |
|  | *OF16328* | CN | *ASE24* |
|  | *OF16890* | NL | *ASE25* |
|  | *OF16910* | NL | *ASE26* |
|  | *OF16912* | N | *ASE27* |
|  | *OF17398* | NL | *ASE28* |
|  | *OF18469* | NL | *ASE29* |
|  | *OF18472* | NL | *ASE30* |
|  | *OF18476* | NL | *ASE31* |
|  | *OF18480* | NL | *ASE32* |
|  | *OF18481* | NL | *ASE33* |
|  | *OF18568* | CNL | *ASE34* |
|  | *OF19245* | NL | *ASE35* |
| **Continued** |  |  |  |
| **Species** | **Gene ID** | **Classification** | **renaming** |
| *A. setaceus* | *OF06161* | CN | *ASE36* |
|  | *OF06756* | N | *ASE37* |
|  | *OF07688* | NL | *ASE38* |
|  | *OF21387* | CNL | *ASE39* |
|  | *OF22822* | CN | *ASE40* |
|  | *OF22823* | CNL | *ASE41* |
|  | *OF22828* | CN | *ASE42* |
|  | *OF22833* | N | *ASE43* |
|  | *OF23889* | CNL | *ASE44* |
|  | *OF24164* | NL | *ASE45* |
|  | *OF04302* | NL | *ASE46* |
|  | *OF04304* | NL | *ASE47* |
|  | *OF04306* | NL | *ASE48* |
|  | *OF04307* | NL | *ASE49* |
|  | *OF04309* | N | *ASE50* |
|  | *OF04311* | N | *ASE51* |
|  | *OF04390* | CNL | *ASE52* |
|  | *OF04392* | CNL | *ASE53* |
|  | *OF04471* | NL | *ASE54* |
|  | *OF04473* | NL | *ASE55* |
|  | *OF05315* | N | *ASE56* |
|  | *OF05316* | NL | *ASE57* |
|  | *OF19905* | RNL | *ASE58* |
|  | *OF19907* | RNL | *ASE59* |
|  | *OF10609* | NL | *ASE60* |
|  | *OF10706* | N | *ASE61* |
|  | *OF11945* | NL | *ASE62* |
|  | *OF12113* | NL | *ASE63* |

Table S3. Adjacent pairs of NLR genes pairs in three species

| **Species** | **Gene pairs** | | **Type** |  |
| --- | --- | --- | --- | --- |
| *A. officinalis* | *Aof21103* | *Aof21105* | head-to-tail |  |
|  | *Aof27699* | *Aof27700* | head-to-tail |  |
|  | *Aof28612* | *Aof28615* | head-to-tail |  |
|  | *Aof31148* | *Aof31150* | head-to-tail |  |
| *A. kiusianus* | *K1p1ch01g28595* | *K1p1ch01g28596* | head-to-tail |  |
|  | *K1p1ch03g01635* | *K1p1ch03g01636* | head-to-tail |  |
|  | *K1p1ch04g21098* | *K1p1ch04g21101* | head-to-head |  |
|  | *K1p1ch05g06225* | *K1p1ch05g06230* | tail-to-tail |  |
|  | *K1p1ch05g06225* | *K1p1ch05g06232* | head-to-tail |  |
|  | *K1p1ch05g06230* | *K1p1ch05g06232* | head-to-head |  |
|  | *K1p1ch06g35722* | *K1p1ch06g35725* | head-to-tail |  |
|  | *K1p1ch06g35768* | *K1p1ch06g35769* | head-to-tail |  |
|  | *K1p1ch06g35768* | *K1p1ch06g35771* | head-to-tail |  |
|  | *K1p1ch06g35768* | *K1p1ch06g35775* | head-to-tail |  |
|  | *K1p1ch06g35769* | *K1p1ch06g35771* | head-to-tail |  |
|  | *K1p1ch06g35769* | *K1p1ch06g35775* | head-to-tail |  |
|  | *K1p1ch06g35769* | *K1p1ch06g35778* | head-to-tail |  |
|  | *K1p1ch06g35771* | *K1p1ch06g35775* | head-to-tail |  |
|  | *K1p1ch06g35771* | *K1p1ch06g35778* | head-to-tail |  |
|  | *K1p1ch06g35775* | *K1p1ch06g35778* | head-to-tail |  |
|  | *K1p1ch06g35927* | *K1p1ch06g35928* | head-to-tail |  |
|  | *K1p1ch07g46492* | *K1p1ch07g46493* | head-to-tail |  |
|  | *K1p1ch08g18532* | *K1p1ch08g18536* | tail-to-tail |  |
|  | *K1p1ch09g57372* | *K1p1ch09g57377* | head-to-tail |  |
|  | *K1p1ch09g57372* | *K1p1ch09g57376* | head-to-tail |  |
|  | *K1p1ch09g57372* | *K1p1ch09g57378* | head-to-tail |  |
|  | *K1p1ch09g57372* | *K1p1ch09g57379* | head-to-tail |  |
|  | *K1p1ch09g57377* | *K1p1ch09g57376* | head-to-tail |  |
|  | *K1p1ch09g57377* | *K1p1ch09g57378* | head-to-tail |  |
|  | *K1p1ch09g57377* | *K1p1ch09g57379* | head-to-tail |  |
|  | *K1p1ch09g57376* | *K1p1ch09g57378* | head-to-tail |  |
|  | *K1p1ch09g57376* | *K1p1ch09g57379* | head-to-tail |  |
|  | *K1p1ch09g57376* | *K1p1ch09g57386* | head-to-tail |  |
|  | *K1p1ch09g57378* | *K1p1ch09g57379* | head-to-tail |  |
|  | *K1p1ch09g57378* | *K1p1ch09g57386* | head-to-tail |  |
|  | *K1p1ch09g57379* | *K1p1ch09g57386* | head-to-tail |  |
|  | *K1p1ch01g28595* | *K1p1ch01g28596* | head-to-tail |  |
| *A. setaceus* | *OF12551* | *OF12552* | head-to-tail |  |
|  | *OF12552* | *OF12553* | head-to-tail |  |
| **Continued** |  |  |  |  |
| **Species** | **Gene pairs** | **Type** | |  |
|  | *OF12552* | *OF12557* | head-to-tail |  |
|  | *OF12553* | *OF12553* | head-to-tail |  |
|  | *OF12796* | *OF12557* | head-to-tail |  |
|  | *OF15791* | *OF12557* | head-to-tail |  |
|  | *OF15901* | *OF12797* | head-to-tail |  |
|  | *OF24611* | *OF15793* | head-to-tail |  |
|  | *OF27419* | *OF15902* | head-to-tail |  |
|  | *OF28280* | *OF24612* | head-to-tail |  |
|  | *OF02990* | *OF27421* | head-to-tail |  |
|  | *OF16910* | *OF28281* | head-to-tail |  |
|  | *OF18469* | *OF02992* | head-to-tail |  |
|  | *OF18469* | *OF16912* | tail-to-tail |  |
|  | *OF18472* | *OF18472* | head-to-tail |  |
|  | *OF18472* | *OF18476* | head-to-head |  |
|  | *OF18472* | *OF18476* | head-to-head |  |
|  | *OF18476* | *OF18480* | head-to-head |  |
|  | *OF18476* | *OF18481* | head-to-head |  |
|  | *OF18480* | *OF18480* | head-to-tail |  |
|  | *OF22822* | *OF18481* | head-to-tail |  |
|  | *OF22822* | *OF18481* | head-to-tail |  |
|  | *OF22823* | *OF22823* | head-to-tail |  |
| *A. setaceus* | *OF22828* | *OF22828* | head-to-tail |  |
|  | *OF04302* | *OF22828* | head-to-tail |  |
|  | *OF04302* | *OF22833* | head-to-tail |  |
|  | *OF04302* | *OF04304* | head-to-tail |  |
|  | *OF04302* | *OF04306* | head-to-head |  |
|  | *OF04302* | *OF04307* | head-to-tail |  |
|  | *OF04304* | *OF04309* | head-to-tail |  |
|  | *OF04304* | *OF04311* | head-to-tail |  |
|  | *OF04304* | *OF04306* | head-to-head |  |
|  | *OF04304* | *OF04307* | head-to-tail |  |
|  | *OF04306* | *OF04309* | head-to-tail |  |
|  | *OF04306* | *OF04311* | head-to-tail |  |
|  | *OF04306* | *OF04307* | tail-to-tail |  |
|  | *OF04307* | *OF04309* | tail-to-tail |  |
|  | *OF04307* | *OF04311* | tail-to-tail |  |
|  | *OF04309* | *OF04309* | head-to-tail |  |
|  | *OF04390* | *OF04392* | head-to-tail |  |
|  | *OF04471* | *OF04473* | head-to-tail |  |
| **Continued** |  |  |  |  |
| **Species** | **Gene pairs** | **Type** | |  |
| *A. setaceus* | *OF05315* | *OF05316* | head-to-tail |  |
|  | *OF19905* | *OF19907* | head-to-tail |  |

Table S4. The estimated ages of the duplicated NLR gene pairs in *A. officinalis* and *A. setaceus*

| **Duplicated gene pairs** | | **Ka** | **Ks** | **ω** | **Time (MYA)** |
| --- | --- | --- | --- | --- | --- |
| *Aof00622* | *OF03029* | 0.22 | 0.39 | 0.55 | 30.31 |
| *Aof06564* | *OF04471* | 0.13 | 0.26 | 0.51 | 20.28 |
| *Aof07053* | *OF22823* | 0.03 | 0.20 | 0.16 | 15.51 |
| *Aof07990* | *OF21387* | 0.04 | 0.09 | 0.40 | 7.17 |
| *Aof11385* | *OF12797* | 0.10 | 0.17 | 0.60 | 13.38 |
| *Aof11395* | *OF12797* | 0.10 | 0.16 | 0.62 | 12.42 |
| *Aof15186* | *OF06161* | 0.14 | 0.22 | 0.62 | 16.89 |
| *Aof22120* | *OF10706* | 0.14 | 0.24 | 0.56 | 18.81 |
| *Aof23470* | *OF27419* | 0.09 | 0.15 | 0.57 | 11.73 |
| *Aof27699* | *OF24612* | 0.04 | 0.13 | 0.32 | 10.36 |
| *Aof27700* | *OF24612* | 0.05 | 0.14 | 0.36 | 11.12 |
| *Aof28500* | *OF18568* | 0.03 | 0.13 | 0.23 | 10.09 |
| *Aof28615* | *OF18480* | 0.02 | 0.13 | 0.14 | 9.89 |
| *Aof31148* | *OF16910* | 0.15 | 0.33 | 0.45 | 25.38 |
| *Aof31150* | *OF16910* | 0.12 | 0.37 | 0.33 | 28.36 |
| *Aof31753* | *OF16328* | 0.15 | 0.32 | 0.47 | 24.76 |
